# Determination of per- and polyfluoroalkyl compounds in paper recycling grades using ultra-high-performance liquid chromatography–high-resolution mass spectrometry

**DOI:** 10.1007/s11356-024-33250-9

**Published:** 2024-04-11

**Authors:** Nondumiso Nomonde Mofokeng, Lawrence Mzukisi Madikizela, Ineke Tiggelman, Edmond Sanganyado, Luke Chimuka

**Affiliations:** 1 Molecular Sciences Institute, School of Chemistry, University of the Witwatersrand, 1 Jan Smuts Ave, Braamfontein, Johannesburg, 2000 South Africa; 2 Mpact Operations Pty (Ltd), Innovation, Research & Development, Devon Valley Road, Stellenbosch, 7600 South Africa; 3 Institute for Nanotechnology and Water Sustainability, College of Science, Engineering and Technology, University of South Africa, Florida Science Campus, 28 Pioneer Ave, Roodepoort, Johannesburg, 1709 South Africa; 4 Department of Applied Sciences, Northumbria University, Newcastle upon Tyne, NE1 8ST UK

**Keywords:** PFAS, Recycled paper, Pollutant prevalence, Ultra-trace analysis, Waste management, Analytical method

## Abstract

Globally, per- and polyfluoroalkyl substances (PFAS)–related research on paper products has focused on food packaging with less consideration on the presence of PFAS at different stages of the paper recycling chain. This study analysed the prevalence of PFAS in paper grades used for the manufacture of recycled paperboard. The presence of PFAS was attributed to the use of PFAS-containing additives, consumer usage, exposure to packed goods as well as contamination during mingling, sorting, collection, and recovery of paper recycling material. Q Orbitrap mass spectrometry was used to analyse the paper samples after accelerated solvent extraction and solid phase extraction. The distribution and possible propagation of 22 PFAS were determined in pre-consumer, retail and post-consumer paper products. Post-consumer samples had the highest combined average concentration (ΣPFAS) at 213 ng/g, while the ΣPFAS in retail (159 ng/g) and pre-consumer samples (121 ng/g) was detected at lower concentrations. This study showed that waste collection and recycling protocols may influence PFAS propagation and that measures must be developed to minimise and possibly eliminate exposure opportunities.

**Supplementary information:**

The online version contains supplementary material available at 10.1007/s11356-024-33250-9.

## Introduction

Over the past 5 years, more than 50 million tons of paper products were recycled annually, with almost half of these being used to manufacture corrugated boxes (World Economic Forum 2022). In the same period, the global paper recycling rate was 68% (Van Ewijk et al. 2021), with South Africa having an average five-year paper recovery rate of 67% (Paper Manufacturers Association of South Africa 2022). The growing emphasis on circular economy and thereby paper recycling (Semple et al. 2022) means that it is imperative to investigate how potentially toxic substances such as per- and polyfluoroalkyl substances (PFAS) propagate across the paper recycling value chain. PFAS may be introduced during the initial manufacture of packaging if they are applied as moisture and grease repellents (Schultes et al. 2019). In addition to the manufacturing stage, PFAS exposure may occur during transportation, storage, use, and disposal of paper products as well as from the mingling of recovered paper with other waste materials during collection, sorting, and repulping (Reinhart et al. 2023). In addition, PFAS present in the soil, air, and water (Solan et al. 2023) may interact with paper destined for recycling. PFAS have been shown to be present at waste disposal sites and landfills (Helmer et al. 2022) and can thereby be present at recycling facilities where recycling includes paper recovered from solid waste disposal sites and landfills. The true contribution of PFAS from paper recycling processes is currently not fully understood in developed and developing countries (Geueke et al. 2018).

PFAS, often referred to as “forever chemicals”, are classified as very persistent, bioaccumulative, and toxic (Cousins et al. 2020; Chambers et al. 2021; Liu and Sun 2021; Brunn et al. 2023). Long-chain PFAS, which typically contain fluoroalkyl chains of six or more carbons (Chambers et al. 2021; Baldwin et al. 2023), possess extremely stable carbon–fluorine bonds, making them more resistant to degradation (Chambers et al. 2021). These longer-chain PFAS have increased hydrophobicity which promotes their retention in paper’s organic, cellulose lattice (Liu and Sun 2021). Initial research into PFAS suggested that PFAS compounds with alkyl chain lengths of five carbons or less were significantly less bioaccumulative and less toxic (Quinete et al. 2010). This led manufacturers to a shift towards shorter-chain PFAS which have now also been shown to be very stable (Brendel et al. 2018), toxic (Lu et al. 2017; Solan et al. 2023) and persistent in the environment (Vierke et al. 2014; Brendel et al. 2018). These short-chain PFAS often have a higher mobility in aqueous environments owing to their higher solubility (Vierke et al. 2014). Since paper manufacturing is associated with high water use (Blanco et al. 2013), PFAS may easily circulate during the manufacture of recycled paper.

The analysis of PFAS in different paper grades used in the manufacture of recycled paper is important to further understand the global occurrence of PFAS. Globally, PFAS-related research on paper products has been focused on food packaging (Trier et al. 2011; Rosenmai et al. 2016; Curtzwiler et al. 2021; Timshina et al. 2021) with less focus on other grades of paper such as newspapers, magazines and packaging used for non-food applications (Glüge et al. 2020). This study aimed to fill this gap in understanding the possible presence of PFAS in different paper recycling grades used to manufacture recycled paperboard. This investigation is crucial as it addresses the challenges of paper recycling processes by pinpointing the possibility of PFAS circulation through the mechanisms employed in solid waste management. The findings of the present study are important for showcasing a need to develop measures that could assist in minimising and eliminating PFAS exposure opportunities.

## Materials and methods

### Chemicals and buffers

The chemicals and buffers used were LC–MS grade methanol (Anatech, Cape Town, South Africa), water (Merck, Johannesburg, South Africa), ammonium formate (Merck, Johannesburg, South Africa), HPLC grade toluene (Merck, Johannesburg) and analytical grade ammonium hydroxide (Merck, Johannesburg, South Africa). Certified reference standards made up in methanol were obtained from AccuStandard (Stargate, Johannesburg, South Africa), the details of which are shown in Table S1. The mixture standard contained both short- and long-chain per- and polyfluorocarboxylic acids (PFCAs) and sulfonic acids (PFSAs). Perfluorohexanesulfonic acid (PFHxS) and perfluorooctanesulfonic acid (PFOS) were present as linear and branched isomers with both isomers considered in the calibration standards and quantification. The calibration standards were made up in methanol in two concentration ranges, from 0.2 to 120 µg/L and from 30 to 450 µg/L.

### Sampling

**Fig. 1:**
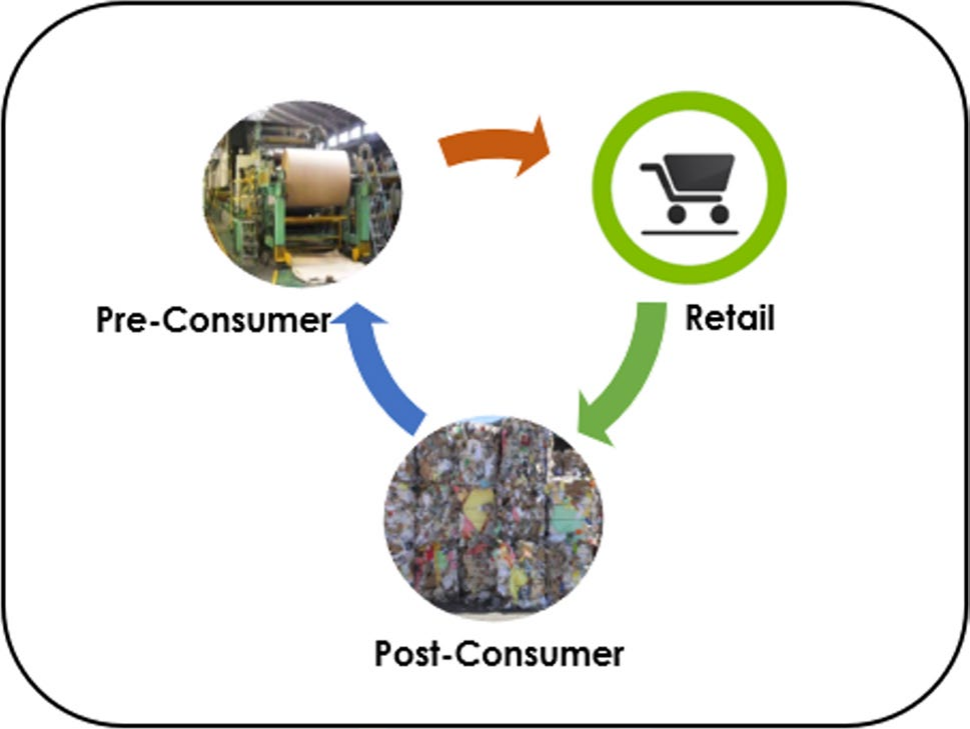
Sample overview

Thirty-nine paper samples were collected from Cape Town, South Africa, and consisted of various grades of paper used for recycling (Fig. 1; Table S2). The pre-consumer and retail samples consisted only of boards, while the post-consumer samples consisted of paper grades typically used in the manufacture of recycled boards. These included cartonboards, newspapers, corrugated boards (boxes), office paper and magazines. The pre-consumer samples were sourced directly from paper mills and paper conversion sites, whereas retail samples were collected from South African grocery stores. Post-consumer samples were collected at recycling facilities, solid waste disposal sites, household waste and informal waste pickers.

### Sample preparation

Paper samples were prepared in triplicate (1 g each), followed by accelerated solvent extraction (ASE) using a Dionex 350 ASE system (Anatech, Cape Town, South Africa). The extraction method was optimised for ethanol:methanol solvent combination (Table S3), with the final optimised conditions being a 3:2 ethanol:methanol combination at 70 °C oven temperature for three cycles at 12 min each (MacLennan et al. 2019). After extraction, the sample was evaporated to 1 mL under nitrogen at 40 °C using a Biotage TurboVap (Anatech, Cape Town, South Africa) in preparation for SPE clean-up. The SPE process was based on weak ionic exchange (WAX) mechanism. The SPE steps were guided by the United States (US) Environmental Protection Agency (EPA) Draft Method 1633, EPA Method 533 and Barola et al. (2020) (Wendelken and EPA 2019; Barola et al. 2020; EPA 2021). For this study, 500 mg sorbent bed/6 mL Enviro-Clean WAX SPE cartridges (Stargate Scientific, Johannesburg, South Africa) were used. The cartridges were mounted on a SPE manifold (Stargate Scientific, Johannesburg, South Africa) and operated under vacuum using a DryVac 400 vacuum pump (Air & Vacuum Technologies, Johannesburg, South Africa). The SPE was initiated by conditioning the cartridges with 3 mL of methanol followed with 5 mL of 0.1 M ammonium formate buffer. This was followed by loading 500 µL of sample and elution of analytes using 6 mL of 1% methanolic ammonium hydroxide. The eluted compounds were concentrated to 1 mL under 40 °C nitrogen and filtered into a polypropylene vial with 0.22-µm filter (Stargate Scientific, Johannesburg, South Africa) before analysis by UHPLC-HRMS.

### Instrumentation

High-resolution LC–MS analysis was achieved using a Thermo Scientific Vanquish UHPLC^+^ Q Exactive Focus Orbitrap (Anatech, Cape Town, South Africa). A Hypersil Gold aQ 100 mm × 2.1 mm × 1.9 µm column and Hypersil Gold 50 mm × 3 mm × 1.9 µm delay column (Anatech, Cape Town, South Africa) were used at 35 °C. The autosampler temperature was set to 7 °C. The instrument operating method was adapted from Zhu and Walker (2020), Silva et al. (2021) and Curtzwiler et al. (2021) with the final optimised conditions consisting of solvent A as 0.1% formic acid in water and 10 mM ammonium formate in methanol as solvent B (Zhu and Walker 2020; Silva et al. 2021; Curtzwiler et al. 2021). The initial flowrate was 0.400 mL/min in 30% solvent B which increased to 100% solvent B at 13 min (at 1 mL/min), held until 17 min, before returning to 30% solvent B at 21.1 min (Table S4). The acquisition mode was full scan with data-dependent acquisition (ddMS^2^) in negative mode. The PFAS transitions and retention times are tabled in Table S5.

### Quality assurance

PFAS are ubiquitous compounds (Evich et al. 2022) and are used extensively in analytical chemistry laboratory items such as PTFE-lined reagent bottle caps, O-rings, septa linings and syringe filters. It was therefore important to investigate latent PFAS contamination in the PFAS analysis workflow. The necessary steps were included to measure the possible contamination from sample collection (Table S6) and the UHPLC-HRMS system (Table S7). Preparation blanks were analysed to subtract the analyte concentrations found in the blanks from the detected concentrations in the samples (Table S8). Additionally, a stringent washing and cleaning protocol was established using toluene, 1% methanolic ammonium hydroxide and methanol. The target compounds absent in the preparation blanks were quantified using external calibration quantification, whereas those found to be present were semi-quantitatively determined by subtracting the preparation blank from the detected concentration. PFBA, PFPeA, PFDoA, PFTeDA, PFBS, PFOS, PFNS, PFDS and 4:2FTS were identified as system contaminants and were semi-quantitatively determined. To evaluate the external calibration curves, the coefficient of determination (*R*^2^) was used as an indicator of linearity. The limit of detection (LOD) and limit of quantification (LOQ), which were used as sensitivity indicators, were measured at signal-to-noise ratios of 3 and 10, respectively. Relative recovery and %RSD were used to evaluate the accuracy and precision of the method, respectively. Virgin, unconverted, unprinted paper and paperboard samples were used to evaluate the method performance by spiking the samples at three different concentrations in triplicate. The data was corrected for the relative recoveries obtained by considering the unspiked paper and paperboard samples. Due to the use of external standards and expected variability, the quantified concentrations were reported with their associated uncertainty using Eq. (1):

1 $$U = \sqrt{{s}^{2}+ {u}_{{\text{matrix}}}^{2}+{u}_{{\text{recovery}}}^{2}+{u}_{{\text{prep}}.\mathrm{ blanks}}^{2}}$$

where *s*^2^ is the variance of the sample, *u*_matrix_ is the uncertainty of the sample repeatability due to matrix effects, *u*_recovery_ is the uncertainty of the recovery of the target compound and *u*_prep. blank_ is the uncertainty related to the preparation blanks (Rasul et al. 2017; Ellison and Williams 2012). The equations related to each term are shown in equations (S1) to (S4).

### Data analysis

Instrument data analysis was performed using Thermo Scientific Xcalibur™. Further data analysis was performed using Microsoft Excel, MetaboAnalyst 5.0 and TIBCO Statistica 14.0. The statistical analysis aimed to identify possible correlations between the target analytes. TIBCO Statistica 14.0. was used to determine if the data followed normal or non-normal distributions. For normally distributed data, the Pearson correlation coefficient would be employed, while the Spearman rank correlation, a non-parametric correlation, would be used for non-normal data that contains outliers (Leon 1998). Once established, the Spearman correlation was plotted using MetaboAnalyst 5.0. The Spearman rank equation is defined as

2 $${r}_{s}=1- \frac{6\sum {d}^{2}}{n({n}^{2}-1)}$$

where the Spearman rank coefficient *r*_*s*_ is calculated by determining the sum of the squared differences in distance (*d*^2^) for *n* observations. A value of *r*_*s*_ = 1 indicates a linear positive relationship, while a value *of r*_*s*_ < 0 indicates a negative relationship between two variables, where an increase in the value of one variable would be associated with a decrease in the second variable (Leon 1998). Hierarchical clustering analysis was performed in conjunction with Spearman correlation. Hierarchical clustering allows for similarity analysis of one sample to the entire dataset and can provide insight into any heterogeneity or homogeneity present in different groups (Kumar et al. 2014).

## Results and discussion

### Method validation

The *R*^2^ of the calibration curves ranged from 0.98 for PFHpA to 0.99 for PFDS, indicating acceptable overall linearity of the target compounds (Table S9). The relative recoveries of paper and board were determined by spiking samples at 10, 20 and 100 ng/g; 20, 40 and 200 ng/g; and 40, 80 and 400 ng/g, depending on the concentration present in the standard mixture. Relative recoveries ranged from 55% for PFHxA to 139% for PFOA (except for 6:2 FTS) (Table S9). For the target PFAS investigated, 19 of the 22 fell within the generally accepted range of 70–130% (Srivastava et al. 2022). PFHxA (10 ng/g paperboard spike), PFOA (10 ng/g paperboard spike) and 6:2 FTS showed poor recovery. This may have been due to the lack of isotopically labelled internal standards that would have likely corrected any matrix effects that would have resulted in changes in ionisation. According to Harris et al. (2022) and Rawn et al. (2022), the use of isotopically labelled internal standards is critical in ensuring absolute quantification and correcting for matrix effects (Rawn et al. 2022; Harris et al. 2022). Other factors, such as SPE interaction, the influence of the buffer solvent system, interaction with other PFAS compounds present and salts, ions and organic matter in the paper samples, may have further influenced the recovery of these target PFAS (Zabaleta et al. 2016; Kaiser et al. 2020; Harris et al. 2022). The %RSD for the relative recoveries ranged from 1.0 to 9.8%, indicating acceptable precision (Srivastava et al. 2022). The method LODs and LOQs were found to be generally lower for the compounds present in the lower concentration in the calibration standard mix with LODs ranging from 1.47 pg/g for PFDA to 10.1 pg/g for PFUnA for compounds present as 2 µg/mL in methanol (Table S9). For the compounds present in larger concentrations, the LODs ranged from 11.2 pg/g for PFPeA to 53.1 pg/g for PFBA. In addition to differences in carbon chain lengths and chemical structures, the stability and possible interactions of the different PFAS compounds in methanol may also have contributed to the differences found in the LOQs and LODs. To date, there have been limited studies on the quantification of paper-based samples using UHPLC Orbitrap HRMS. However, most studies have focused on the use of LC–MS/MS or LC-QTOF-MS (Zabaleta et al. 2016, 2020; Blanco-Zubiaguirre et al. 2021; Boisacq et al. 2023; Vera et al. 2024). The LODs in this study were thus compared with the analysis of PFAS in food matrices and landfill leachate, where the instrument of analysis was an HRMS Q Orbitrap. This study achieved more sensitive detection limits for common PFAS (Rawn et al. 2022; Rehnstam et al. 2023). Rawn et al. (2022) reported LOQs ranging from 18 g/g food for L-PFDS to 12.4 ng/g PFBA food (Rawn et al. 2022). In this study, the limits of quantification ranged from 0.0049 ng/g paper for PFDA to 0.18 ng/g paper for PFBA, further highlighting the sensitivity achieved in this study.

### Occurrence of PFAS

**Fig. 2:**
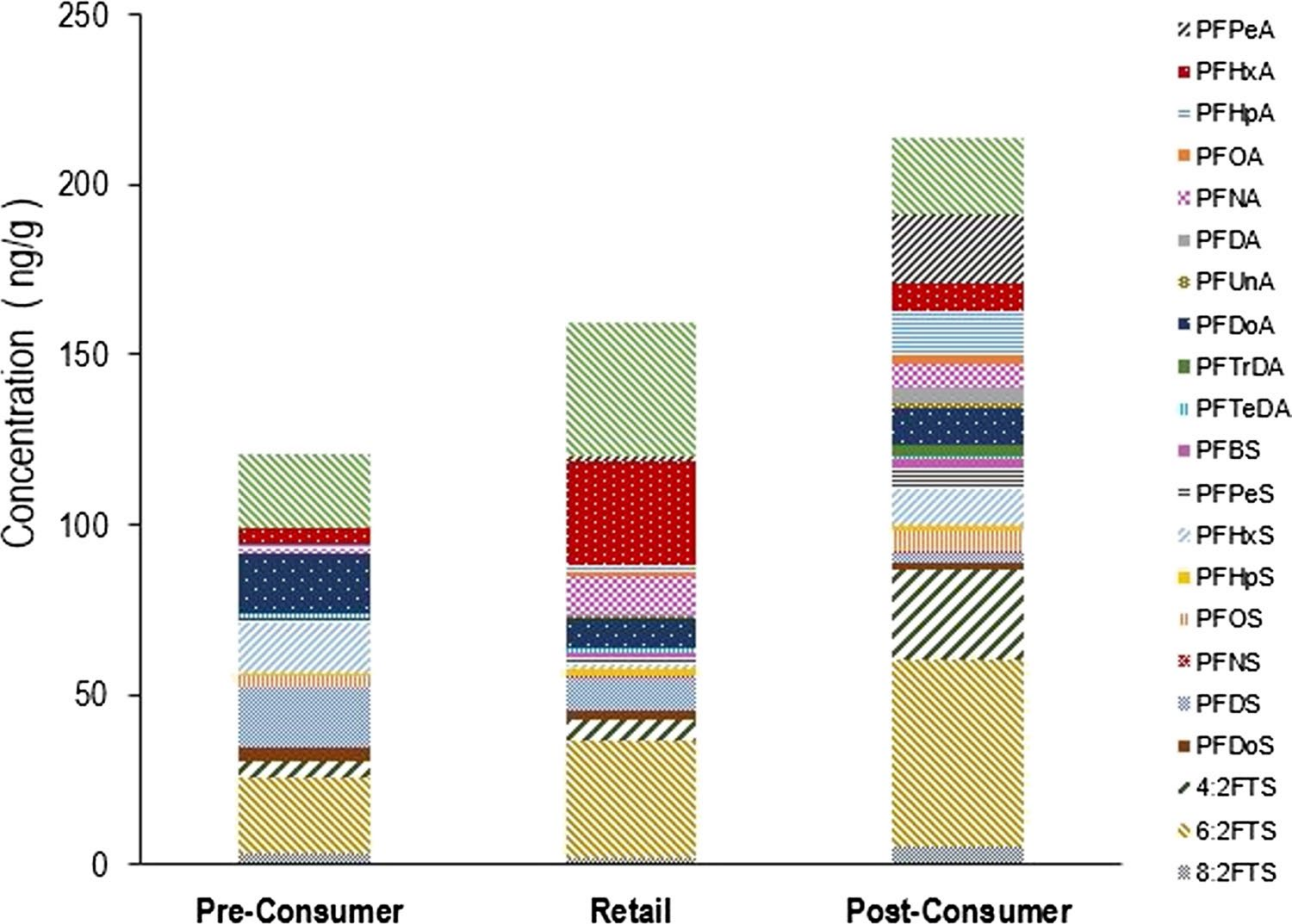
Overview of pre-consumer, retail and post-consumer samples

The combined sum of average concentrations (ΣPFAS) of the 22 analysed PFAS compounds was highest for post-consumer samples (213 ng/g), followed by retail samples (159 ng/g) and pre-consumer samples (121 ng/g), as shown in Fig. 2. Overall, PFAS contamination increased from pre-consumer to post-consumer, with the highest concentration associated with post-consumer samples where recovered material had likely interacted with the environment and possibly mingled with other waste materials. The pre-consumer samples analysed consisted of samples made with a recycled fibre component, whereas the retail and post-consumer samples were expected to contain virgin, mixed and/or recycled fibre. The occurrence of PFAS in these samples was strongly related to PFAS intentionally and/or unintentionally used in manufacturing, retention of PFAS from recycling and, in retail samples, exposure from transport, distribution and packed goods. When looking at the categorical box and whiskers chart plotted using Statistica ® (Table S10), it was further evident that more PFAS were detected in post-consumer samples, with the categorical data being found to statistically follow normal distributions (Fig. S1 to Fig. S22). In terms of the PFAS compounds, 6:2 FTS was the most prevalent target analyte with the highest average concentration of 55.2 ng/g for post-consumer samples, 34.3 ng/g for retail and 22.1 ng/g for pre-consumer samples. This 6-fluoroalkyl chain telomer has become a predominant polymer processing alternative to PFOS and PFOA (Lu et al. 2017) (Schwartz-Narbonne et al. 2023; Solan et al. 2023). In a study by Dueñas-Mas et al. (2023) on food packaging in fast-food restaurants in France, 6:2 FTS and PFHxA were detected in all 47 samples analysed (Dueñas-Mas et al. 2023). It has also been reported in fast-food packaging in the USA (Schaider et al. 2017). This corroborated the prevalence of 6:2 FTS in this study. It is important to note that matrix enhancement effects may have given results that were biased high due to the high 6:2 FTS recovery rate obtained. Along with 6:2 FTS, PFBA, PFHxA, PFDoA and PFTeDA were also frequently detected in the samples. This highlighted the possible circulation of short-chain PFAS. According to Palma et al. (2022) and Rehnstam et al. (2023), shorter-chain PFAS such as PFPeA, PFHxA and PFBA are the main degradation products of both PFOA and PFOS in the environment (Palma et al. 2022). PFPeA and PFHxA are common breakdown products of stain- and grease-proof coatings in food packaging and household products (Liu and Liu 2016).

**Fig. 3:**
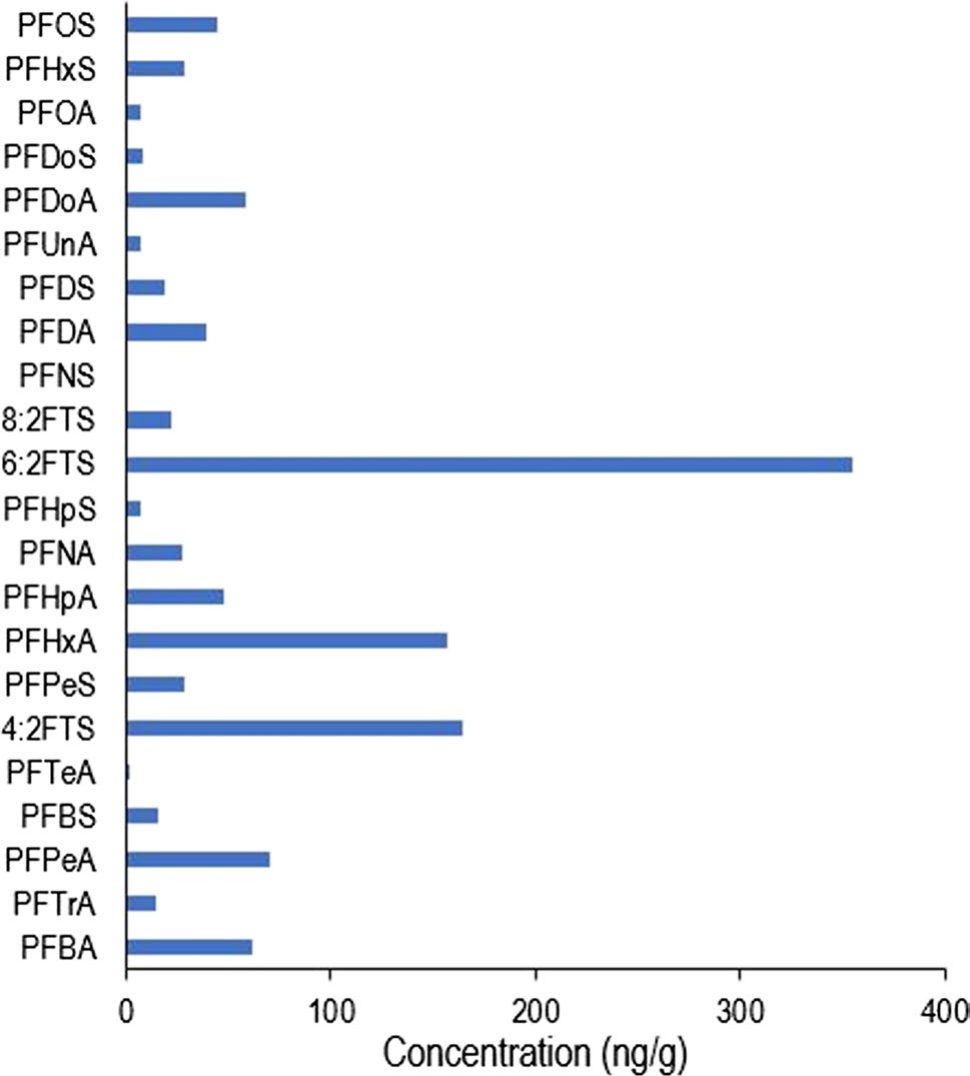
PFAS range

The PFAS range was plotted to compare the ranges of the detected PFAS (Fig. 3). The smallest range was for PFNS from (0–0.04 ng/g), and the largest was for 6:2 FTS (0–354.5 ng/g). The majority of the PFAS compounds detected were below 50 ng/g, with PFDoA (58.3 ± 0.5 ng/g for a recycling facility coffee box), PFPeA (70.4 ± 3.1 ng/g for a waste picker corrugated box), PFHxA (156.9 ± 9.6 ng/g for a retail chocolate box) and 4:2FTS (164.79 ± 7.1 ng/g for the same waste picker corrugated box) all being higher than 50 ng/g. In comparison, the PFAS concentrations reported in Dueñas-Mas et al. (2023) were considerably lower in food packaging with concentrations detected found to be less than 10 ng/g (Dueñas-Mas et al. 2023).

### PFAS profile of samples

**Fig. 4:**
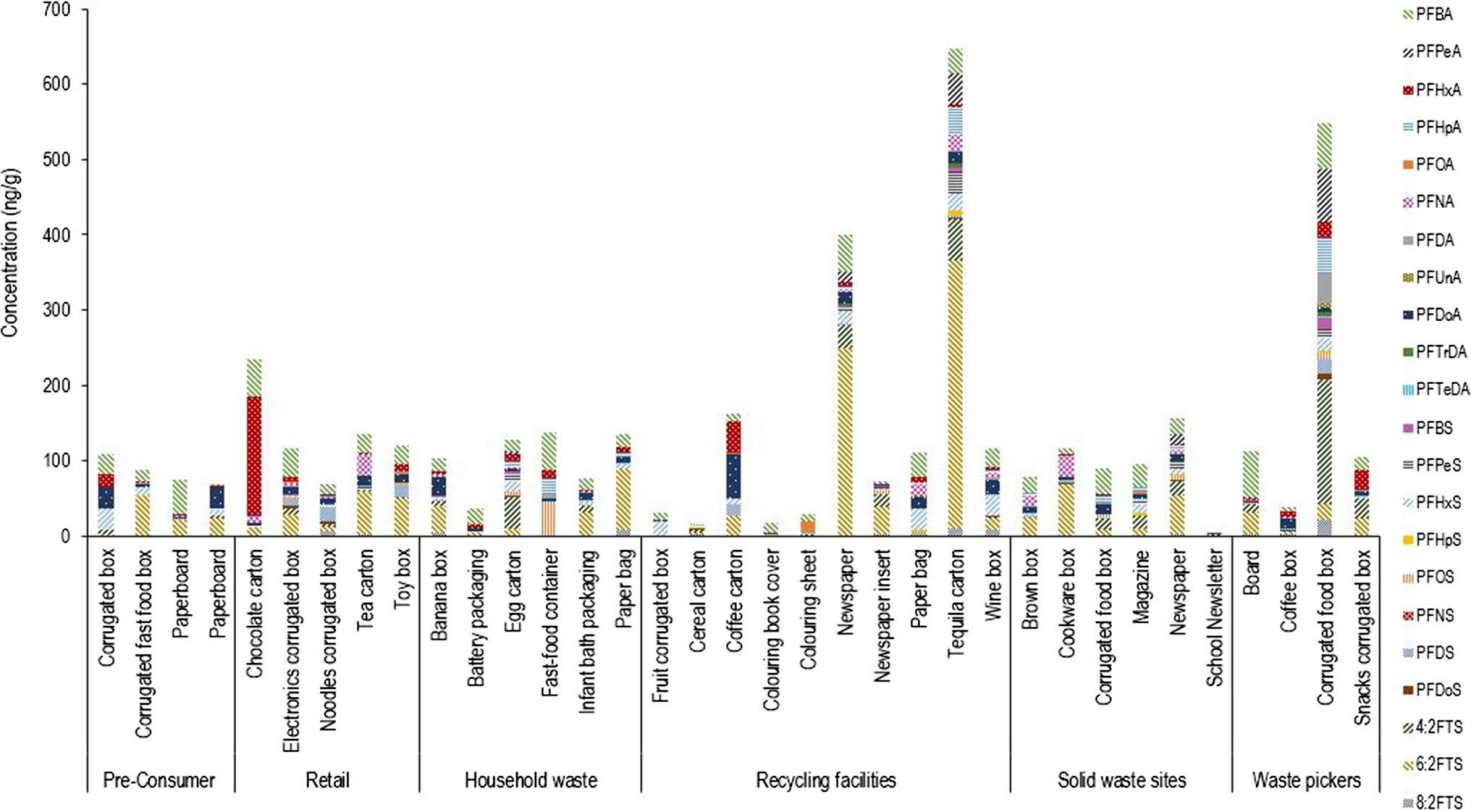
PFAS profile of samples

The PFAS profiles of the samples are plotted in Fig. 4 for the detected concentrations (Tables S11 and S12). When examining the individual PFAS in specific samples, the three highest ΣPFAS were found in post-consumer samples, namely a recycling facility tequila carton (ΣPFAS of 647.9 ng/g), a recycling facility newspaper (ΣPFAS of 400.2 ng/g) and a waste picker corrugated food box (ΣPFAS of 548.0 ng/g). The corrugated food box had the highest number of different PFAS detected with 21 of the 22 quantified PFAS detected. An egg carton collected from household waste was found to contain 18 of the 22 quantified PFAS detected, albeit in smaller combined concentrations (ΣPFAS of 127.6 ng/g) than the waste picker corrugated food box. The lowest concentration of combined PFAS concentration samples was found in a newsletter collected at a solid waste disposal site (ΣPFAS of 2.6 ng/g), a recycling facility cereal carton (ΣPFAS = 14.5 ng/g), a colouring book cover (ΣPFAS = 17.2 ng/g) and a recycling facility corrugated fruit box (ΣPFAS 31.9 ng/g). Among the retail samples, a chocolate carton had the highest combined PFAS concentration (ΣPFAS = 235.1 ng/g) with PFHxA being the dominant PFAS. Across the paper recycling chain, the average ΣPFAS in the corrugated boxes increased from pre-consumer (99 ng/g) to retail (102 ng/g) to post-consumer (145 ng/g). All samples analysed in this study were found to contain PFAS, further demonstrating the prevalence of PFAS compounds. This differed Zabaleta et al. (2020) and other findings related to targeted quantification (Zafeiraki et al. 2014; Zabaleta et al. 2016), where the majority of the food packaging material was found to contain PFAS at very low concentrations or not detected at all. However, it correlated with Dueñas-Mas et al. (2023) and Sapozhnikova et al. (2023). In the study by Sapozhnikova et al. (2023), PFHpA, PFDA, 6:2 fluorotelomer phosphate diester and PFHxS were the most frequently detected PFAS(Sapozhnikova et al. 2023). These comparisons showed that the detection of PFAS may be dependent on the region, circulating PFAS, exposure pathways, analysis protocols and analytical method sensitivity.

**Fig. 5:**
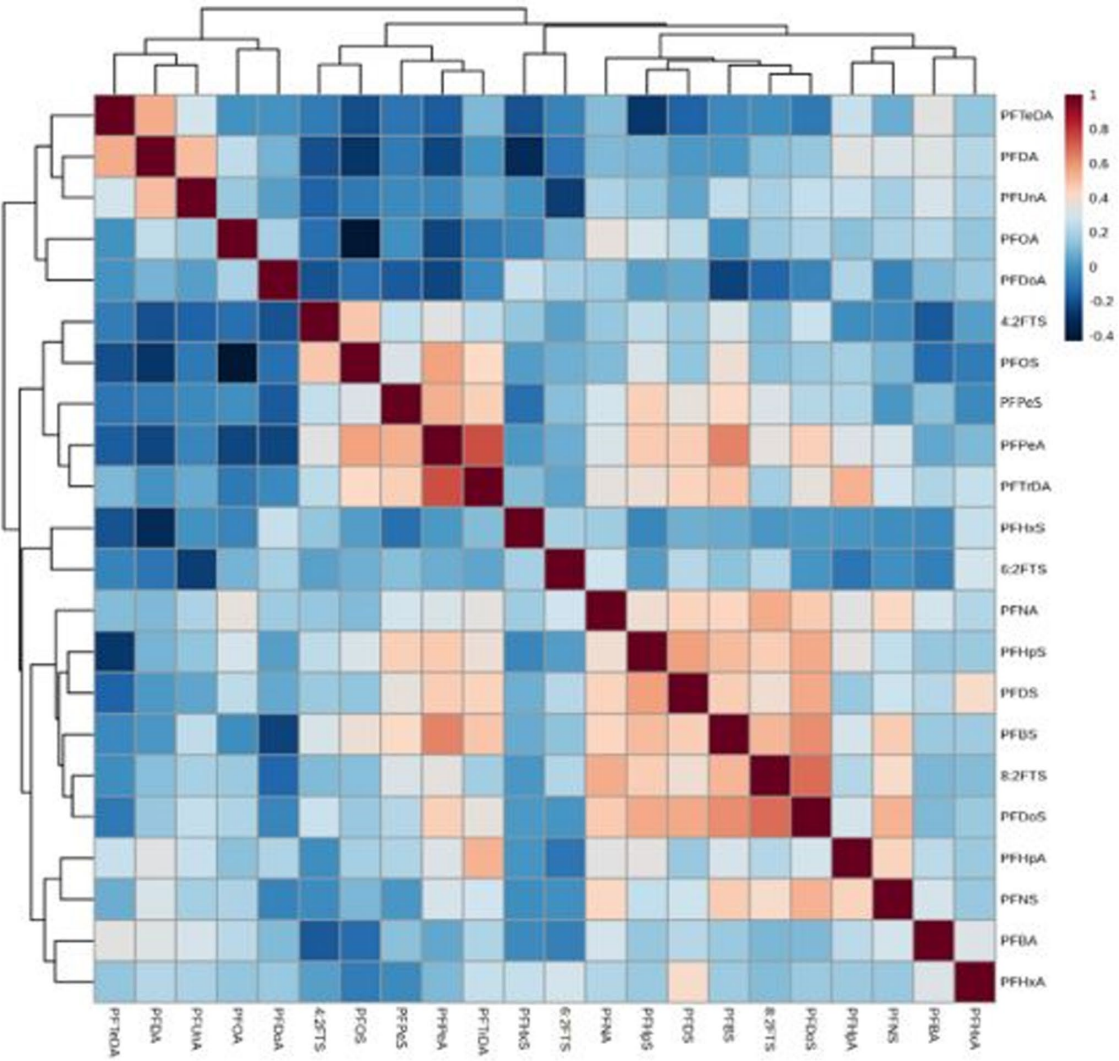
PFAS compound correlation heat map

**Fig. 6:**
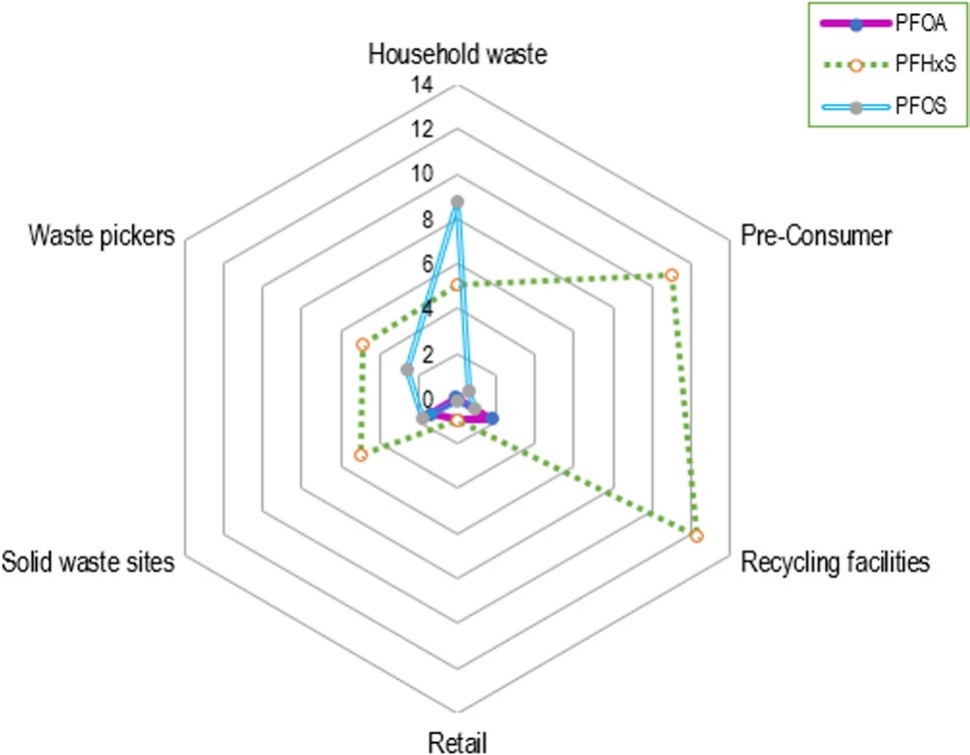
Stockholm-listed PFAS radar chart

The uncategorised data followed non-normal distributions with outliers (Fig. S23). Spearman rank correlation (Eq. (1)) was used as the distance measure for the correlation heat map which was constructed with its associated dendrogram using MetaboAnalyst 5.0. In Fig. 5, a correlation of 1 (shown in dark red) represented a directly proportional relationship and − 0.4 indicated the most negative correlation, shown in dark blue. The correlation between the different PFAS under investigation showed that the strongest correlation was between PFPeA and PFTrDA (0.69) and between 8:2 FTS and PFDoS (0.30). This indicated that the presence of PFPeA was accompanied by the detection of PFTrDA in the same sample. Furthermore, the detection of PFTeDA was associated with the presence of PFDA and PFUnA. The PFAS found to likely co-occur to some extent were PFNA, PFHpS, PFDS, PFBS, 8:2 FTS and PFDoS. Conversely, PFTeDA and PFHpS had a strong negative correlation at − 0.261. A hierarchical clustering dendrogram as shown in Fig. 6 was based on the principle that variables with similar numerical values would also be close to each other in space (Kumar et al. 2014). The dendrogram indicated distinct clustering in the presence of several target PFAS in various papers. For instance, it could be seen that PFTeDA, PFDA, PFUnA, PFOA and PFDoA clustered together when compared to the remaining target analytes. In addition, further clustering was evident between PFTeDA and PFDA with PFUnA compared to PFOA and PFDoA. The investigation of such clustering was beneficial for understanding the detection of numerous types of poly- and perfluorocarboxylic and sulfonic acids in different paper samples and environmental media (Trier et al. 2011; Zabaleta et al. 2020; Bai and Son 2021; Kurwadkar et al. 2022; Bugsel et al. 2022). This further suggested that it would be difficult to isolate a single PFAS compound for targeted removal, for instance, without determining all possible co-occurrences.

### Stockholm-listed PFAS

In 2009, PFOS and its salts were the first PFAS to be listed in the United Nations Stockholm Convention list of persistent organic pollutants. The listing of PFOA followed in 2019, with PFHxS formally listed most recently in 2022 (UNEP 2019a). When looking specifically at the Stockholm Convention–listed PFAS, the highest detection was at 19.9 ± 2.1 ng/g for PFHxS in a pre-consumer corrugated board. PFHxS was the most prevalent PFAS in the Stockholm Convention–listed PFAS, followed by PFOS and PFOA (as shown in Fig. 6). Sapozhnikova et al. (2023) reported that PFHxS was one of the most frequently detected PFAS in their study of globally sourced food packaging (Sapozhnikova et al. 2023). PFHxS is used as a surfactant in various applications, including firefighting foams, metal plating and textiles (Boucher et al. 2019). PFHxS and its related compounds have also been associated with paper production (UNEP 2019b; Langberg et al. 2021), which could explain their detection in this study. In addition, PFHxS has been found to form from perfluoroalkyl sulfonamide derivatives during chlorination and chloramination during water disinfection (Li et al. 2023) and bioaccumulate in the environment (Zhong et al. 2021), which may be another exposure pathway in the paper recycling chain. When examining the other listed PFAS in this study, PFOS was more prominent in the household waste samples with the highest concentration detected in a fast-food container (44.4 ± 1.6 ng/g). PFOA, on the other hand, was less prominent in household waste and pre-consumer samples but was more prevalent in post-consumer solid waste disposal and recycling facility samples, in the colouring sheet sample (14.3 ± 0.7 ng/g) and magazine sample (7.0 ± 1.4 ng/g), respectively. This shows that these longer-chain PFAS of concern may be circulating in the environment and consumer-good value chains. Many developing countries are guided by the Stockholm Convention and the ratification thereof. The co-occurrences shown in this study are therefore important for the Global South as they open avenues for further research that can be used to motivate for legislation that is driven towards the restriction of all PFAS.

## Concluding remarks

The analysis of PFAS using UHPLC-HRMS highlighted the difficulty of PFAS analysis given the ubiquitous nature of PFAS in the samples as well as in laboratory ware, solvents and instrumentation. Blank samples were largely able to account for contamination issues; however, the lack of internal standards meant that the matrix effects could not be accounted for. When comparing the results obtained, it was shown that the combined average concentration of PFAS was lowest in the pre-consumer samples and highest in the post-consumer samples. This study further showed that the co-mingling, sorting and collection protocols may influence PFAS propagation, as the highest PFAS concentrations were detected at post-consumer sites. When considering paper recycling and the circular economy, this study illustrated the need to further understand and investigate activities in the entire paper recycling chain. The initial manufacturing, usage, sorting, collection, disposal, recovery and remanufacturing design protocols for recycled paper are key to ensuring minimal PFAS circulation in the paper recycling chain. Research on different exposure pathways is still required to further unpack PFAS contamination, exposure and propagation to fully evaluate the safety and impact of PFAS in paper recycling.

### Supplementary information

Below is the link to the electronic supplementary material.

Supplementary file1 (DOCX 1834 KB)

## Data Availability

All data generated in this study is available in the article supplementary material.
