# Supplementary material for: Determination of per- and polyfluoroalkyl compounds in paper recycling grades using ultra-high-performance liquid chromatography–high-resolution mass spectrometry
Source: Environ Sci Pollut Res Int. 2024 Apr 11;31(20):30126–36. doi: 10.1007/s11356-024-33250-9 (PMC11058588; doi:10.1007/s11356-024-33250-9)
Supplement: Supplementary file 1 — Supplementary file1 (DOCX 1834 KB) [file 11356_2024_33250_MOESM1_ESM.docx]

**Determination of per- and polyfluoroalkyl compounds in paper recycling grades using ultra high performance liquid chromatography - high resolution mass spectrometry**

Nondumiso N. Mofokeng, Lawrence M. Madikizela, Ineke Tiggelman, Edmond Sanganyado, Luke Chimuka

**SUPPLEMENTARY**

**Table S1** Certified reference standard mixture

| **Compound** | **Chemical formula** | **Compound name** | **Chemical structure** | **CAS #** | **Concentration in standard mixture (µg/mL) in methanol** |
| --- | --- | --- | --- | --- | --- |
| PFBA | C_4_HF_7_O_2_ | Perfluoro-n-butanoic acid | 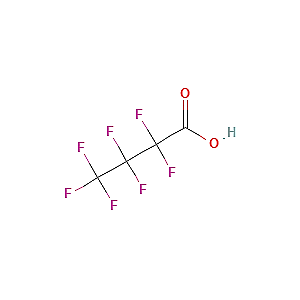 | 375-22-4 | 8 |
| PFPeA | C_5_HF_9_O_2_ | Perfluoro-n-pentanoic acid | 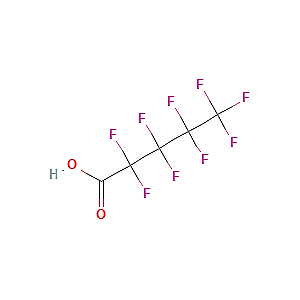 | 2706-90-3 | 4 |
| PFHxA | C_6_HF_11_O_2_ | Perfluoro-n-hexanoic acid | 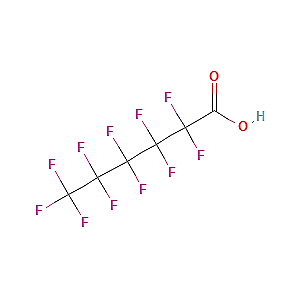 | 307-24-4 | 2 |
| PFHpA | C_7_HF_13_O_2_ | Perfluoro-n-heptanoic acid | 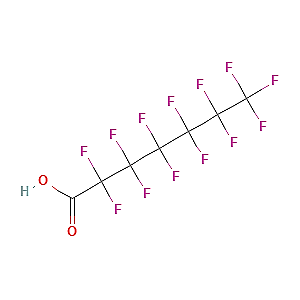 | 375-85-9 | 2 |
| PFOA | C_8_HF_15_O_2_ | Perfluoro-n-octanoic acid | 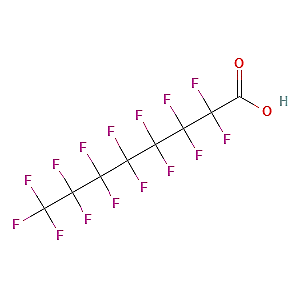 | 335-67-1 | 2 |
| PFNA | C_9_HF_17_O_2_ | Perfluoro-n-nonanoic acid | 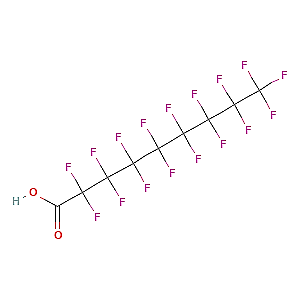 | 375-95-1 | 2 |
| PFDA | C_10_HF_19_O_2_ | Perfluoro-n-decanoic acid | 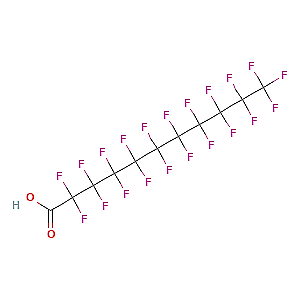 | 335-76-2 | 2 |
| PFUnA | C_11_HF_21_O_2_ | Perfluoro-n-undecanoic acid | 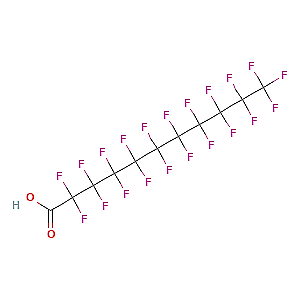 | 2058-94-8 | 2 |
| PFDoA | C_12_HF_23_O_2_ | Perfluoro-n-dodecanoic acid | 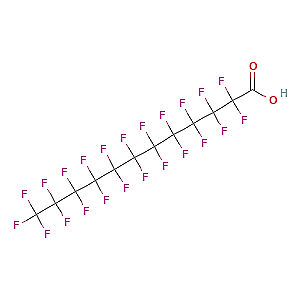 | 307-55-1 | 2 |
| PFTrDA | C_13_HF_25_O_2_ | Perfluoro-n-tridecanoic acid | 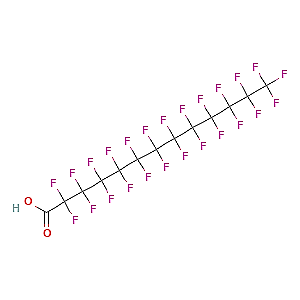 | 72629-94-8 | 2 |
| PFTeDA | C_14_HF_27_O_2_ | Perfluoro-n-tetradecanoic acid | 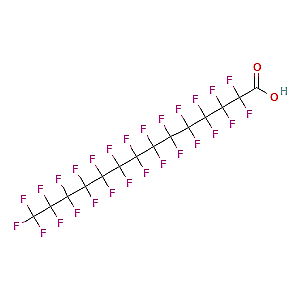 | 376-06-7 | 2 |
| PFBS | C_4_HF_9_O_3_S | Perfluorobutane-1-sulfonic acid | 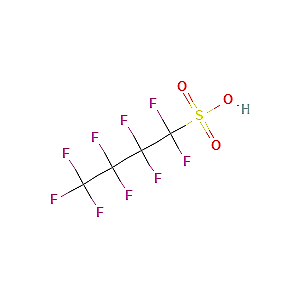 | 375-73-5 | 2 |
| PFPeS | C_5_HF_11_O_3_S | Perfluoropentanesulfonic acid | 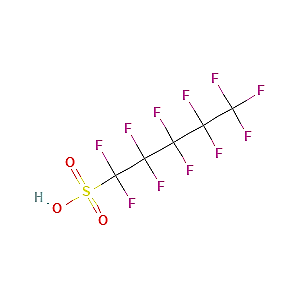 | 2706-91-4 | 2 |
| PFHxS (linear & branched) | C_6_HF_13_O_3_S | Perfluorohexane-1-sulfonic acid | 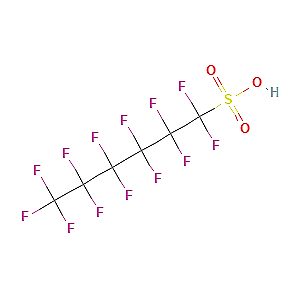 | 355-46-4 | 2 |
| PFHpS | C_7_HF_15_O_3_S | Perfluoroheptanesulfonic acid | 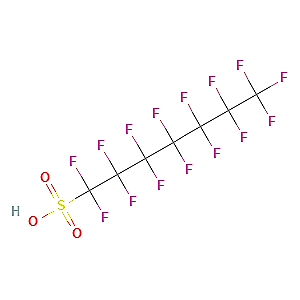 | 375-92-8 | 2 |
| PFOS (linear & branched) | C_8_HF_17_O_3_S | Perfluorooctane-1-sulfonic acid | 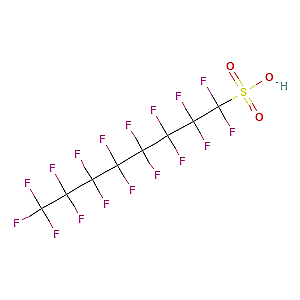 | 1763-23-1 | 2 |
| PFNS | C_9_HF_19_O_3_S | Perfluorononanesulfonic acid | 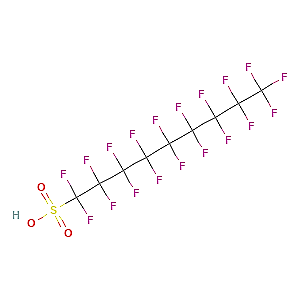 | 68259-12-1 | 2 |
| PFDS | C_10_HF_21_O_3_S | Perfluorodecane-1-sulfonic acid | 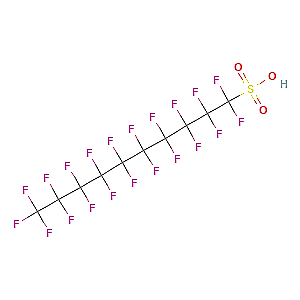 | 335-77-3 | 2 |
| PFDoS | C_12_HF_25_O_3_S | Perfluorododecanesulfonic acid | 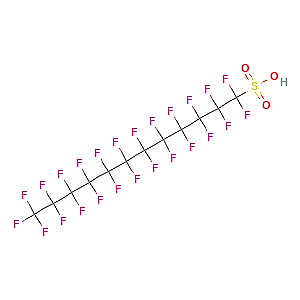 | 79780-39-5 | 2 |
| 4:2 FTS | C_6_H_5_F_9_O_3_S | 1H,1H,2H,2H-Perfluorohexanesulfonic acid | 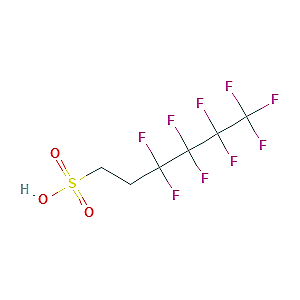 | 757124-72-4 | 8 |
| 6:2 FTS | C_8_H_5_F_13_O_3_S | 1H,1H,2H,2H-Perfluorooctanesulfonic acid | 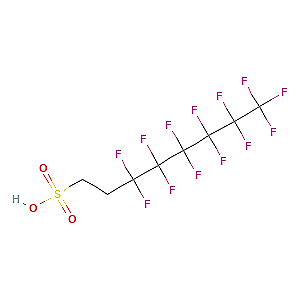 | 27619-97-2 | 8 |
| 8:2 FTS | C_10_H_5_F_17_O_3_S | 1H,1H,2H,2H-Perfluorodecanesulfonic acid | 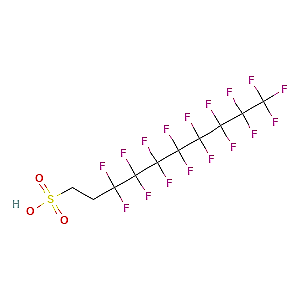 | 39108-34-4 | 8 |

**Table S2** Sample description

| **Site** | **Recycling Paper Grade** | **Number of Samples** |
| --- | --- | --- |
|  |  |  |
| **Mills** | Unconverted paperboard | 3 |
|  |  |  |
| **Corrugators** | Corrugated board | 2 |
|  | |  |
| **Recycling facilities** | Cartonboard | 4 |
|  | Coloured paper | 1 |
|  | Corrugated board | 2 |
|  | Newspaper | 2 |
|  | Black & white office paper | 1 |
|  |  |  |
| **Retail** | Cartonboard | 2 |
|  | Corrugated board | 4 |
|  |  |  |
| **Solid waste disposal sites** | Corrugated board | 3 |
|  | Magazine | 2 |
|  | Newspaper | 2 |
|  | Black & white office paper | 1 |
|  | |  |
| **Waste pickers** | Cartonboard | 1 |
|  | Corrugated board | 3 |
|  |  |  |
| **Household waste** | Cartonboard | 4 |
|  | Corrugated board | 2 |

**ASE Extraction**

A literature survey indicated the two most commonly used solvents in paper-based and solid samples were ethanol. acetonitrile. water and methanol. MacLennan et al. (2019; 2021) used oven temperature 100 °C Static cycle. time 300 s Solvent 80:20 methanol/ acetonitrile with 50 mL volume in 3 cycles 3 whilst Silva et al. (2021) focused ultrasonic liquid extraction (FUSLE) procedure using ethanol. (Trier et al.. 2011) compared extraction of PFAS at room temperatures in ethanol water where 10% ethanol was negligible whilst 50% ethanol and 95% ethanol gave similar results. Ethanol and methanol extractions of 1g of the same unspiked cardboard sample were compared in this study for optimisation of solvent.

**Table S3** Solvent comparison

| **EtOH: MeOH Solvent Ratio** |  | **Concentration (µg/L)** | | | | | | | | | | | | | | |
| --- | --- | --- | --- | --- | --- | --- | --- | --- | --- | --- | --- | --- | --- | --- | --- | --- |
|  | **PFBA** | **PFPeA** | **PFHxA** | **PFHpA** | **PFNA** | **PFDA** | **PFBS** | **PFUnA** | **PFDoA** | **PFTrDA** | **PFTeDA** | **PFHxS** | **PFOS** | **4:2 FTS** | **6:2 FTS** | **8:2 FTS** |
| **1:1** | 23.64 | 4.09 | 4.13 | 7.27 | 2.79 | 0.40 | 0.37 | 0.50 | 6.66 | 0.52 | 0.93 | 0.93 | 0.53 | 6.73 | 29.11 | 0.76 |
| **2:3** | 24.99 | 3.59 | 3.50 | 7.11 | 3.16 | 0.39 | 0.29 | 0.49 | 8.51 | 0.48 | 0.94 | 1.26 | 0.24 | 6.01 | 23.87 | 0.83 |
| **3:2** | 27.79 | 4.37 | 3.67 | 8.58 | 3.99 | 0.40 | 0.39 | 0.49 | 5.15 | 0.54 | 0.91 | 0.70 | 0.65 | 6.93 | 34.74 | 0.76 |
| **%RSD** | 8.30 | 9.81 | 8.59 | 10.53 | 18.66 | 1.38 | 14.13 | 1.47 | 24.86 | 6.16 | 1.38 | 29.27 | 44.85 | 7.40 | 18.60 | 4.92 |

where red- highest value, yellow- middle value, green – lowest value.

From Table S3, the 3:2v/v ethanol: methanol was found to give the highest detected concentrations and was selected as the extraction solvent. The oven temperature was selected as 70°C. based on modification of MacLennan et al. (2019).

**Thermo Scientific Q Exactive Focus UHPLC^+^ Orbitrap HRMS Instrument Parameters**

Solvent A: LC-MS grade water with 10mM Ammonium formate 0.1% formic acid

Solvent B: LC-MS grade methanol

**Table S4** Orbitrap LC-HRMS Flow Gradient

|  | **Time** | **Flow (mL/min)** |  | **%B** | **Curve** |
| --- | --- | --- | --- | --- | --- |
| 1 | 0.00 | 0.400 |  | 30 | 5 |
| 2 | 1.00 | 0.400 |  | 30 | 5 |
| 3 | 13.00 | 1.00 |  | 100 | 3 |
| 4 | 17.00 | 1.00 |  | 100 | 5 |
| 5 | 17.10 | 0.500 |  | 60 | 5 |
| 6 | 21.1 | 0.500 |  | 50 | 5 |
| 7 | 21.10 | 0.500 |  | 30 | 5 |

**Table S5** PFAS Transitions and Retention Times

| **Compound** | **Precursor ion (m/z)** | **Product ions  (m/z)** | **RT (min)** | |
| --- | --- | --- | --- | --- |
| PFBA | 212.9786 | 168.9883; 118.8934 | 2.67 |  |
| PFPeA | 262.9759 | 218.9859; 80.3690 | 4.35 |  |
| PFBS | 298.9456 | 98.9545; 79.9560 | 4.54 |  |
| 4:2 FTS | 326.9744 | 306.9677; 80.9639 | 4.99 |  |
| PFHxA | 312.9730 | 268.9830; 91.0208 | 5.07 |  |
| PFPeS | 348.9398 | 118.9392; 98.9542 | 5.16 |  |
| PFHxS | 398.9387 | 218.9736; 79.9575 | 5.65 |  |
| PFHpA | 362.9695 | 318.9799; 168.9882 | 5.67 |  |
| 6:2 FTS | 426.9674 | 406.9639; 80.9639 | 6.06 |  |
| PFOA | 412.9661 | 168.9884; 118.9911 | 6.11 |  |
| PFHpS | 448.9331 | 112.9842; 75.4488 | 6.19 |  |
| PFNA | 462.9634 | 218.9850; 168.9882 | 6.56 |  |
| PFOS | 498.9714 | 318.9456; 112.9843 | 6.56 |  |
| 8:2 FTS | 526.9605 | 606.955-; 80.0639 | 7.00 |  |
| PFDA | 512.9613 | 468.9704; 218.9854 | 6.99 |  |
| PFNS | 548.9269 | 412.8763; 112.9842 | 7.03 |  |
| PFDS | 598.9269 | 229.9752; 112.3167 | 7.48 |  |
| PFUnA | 562.9562 | 268.9828; 168.9882 | 7.50 |  |
| PFDoA | 612.9535 | 568.9627; 268.9828 | 7.97 |  |
| PFDoS | 699.9198 | 290.9486; 112.9847 | 8.40 |  |
| PFTrDA | 662.9497 | 268.9824; 218.9854 | 8.44 |  |
| PFTeDA | 712.9467 | 268.9824; 218.9854 | 8.89 |  |

**Contamination Considerations for PFAS Workflow**

**Table S6** Other Possible contamination sources: PFAS Concentrations (pg/µL)

|  | **PFBA** | **PFPeA** | **PFHxA** | **PFHpA** | **PFOA** | **PFNA** | **PFDA** | **PFUnA** | **PFDoA** | **PFTrDA** | **PFTeDA** | **PFBS** | **PFPeS** | **PFHxS** | **PFHpS** | **PFOS** | **PFNS** | **PFDS** | **PFDoS** | **4:2 FTS** | **6:2 FTS** | **8:2 FTS** |
| --- | --- | --- | --- | --- | --- | --- | --- | --- | --- | --- | --- | --- | --- | --- | --- | --- | --- | --- | --- | --- | --- | --- |
| **Black PE bag** | 7.840 | <LOD | <LOD | <LOD | <LOD | <LOD | <LOD | <LOD | <LOD | <LOD | 0.396 | 0.492 | <LOD | <LOD | 0.004 | 0.644 | 0.192 | 0.262 | <LOD | 2.938 | <LOD | <LOD |
| **Clear PE** | 8.005 | <LOD | <LOD | <LOD | <LOD | <LOD | <LOD | <LOD | <LOD | <LOD | 0.383 | 0.477 | <LOD | 0.022 | <LOD | 0.747 | 0.205 | 0.281 | <LOD | 0.831 | <LOD | <LOD |
| **Clear PP sachet** | 7.995 | <LOD | <LOD | <LOD | <LOD | <LOD | <LOD | <LOD | <LOD | <LOD | 0.371 | 1.045 | <LOD | 0.08 | 0.041 | 1.168 | 0.238 | 0.565 | <LOD | 2.156 | <LOD | <LOD |
| **Aluminium Foil** | 7.771 | <LOD | <LOD | <LOD | 1.097 | <LOD | <LOD | <LOD | <LOD | <LOD | 0.410 | 0.221 | <LOD | 0.131 | <LOD | 0.556 | 0.193 | 0.254 | <LOD | 0.863 | 16.946 | <LOD |
| **Blender Rinse** | 7.231 | <LOD | <LOD | <LOD | <LOD | <LOD | <LOD | <LOD | <LOD | <LOD | 0.356 | 0.211 | <LOD | <LOD | 0.082 | 2.184 | 0.192 | 0.246 | <LOD | 0.197 | <LOD | <LOD |
| **Nitrile Gloves** | 6.526 | <LOD | <LOD | <LOD | <LOD | <LOD | <LOD | 0.368 | <LOD | <LOD | 0.375 | 0.246 | <LOD | <LOD | 0.072 | 8.26 | 0.205 | 0.234 | <LOD | 0.107 | <LOD | <LOD |

**Table S7** Unfiltered solvent blank concentrations (pg/µL)

| **Solvent Blank (not filtered)** | **PFBA** |  | **PFPeA** | **PFHxA** | **PFHpA** | **PFOA** | **PFNA** | **PFDA** | **PFUnA** | **PFDoA** | **PFTrDA** | **PFTeDA** | **PFBS** | | **PFPeS** | **PFHxS** | **PFHpS** | **PFOS** | **PFNS** | **PFDS** | **PFDoS** | **4:2 FTS** | **6:2 FTS** | **8:2 FTS** |
| --- | --- | --- | --- | --- | --- | --- | --- | --- | --- | --- | --- | --- | --- | --- | --- | --- | --- | --- | --- | --- | --- | --- | --- | --- |
| **Blank 1** | <LOD |  | <LOD | <LOD | <LOD | <LOD | <LOD | <LOD | <LOD | <LOD | <LOD | 0.393 | | 0.351 | <LOD | <LOD | <LOD | 0.532 | 0.193 | 0.256 | <LOD | <LOD | <LOD | <LOD |
| **Blank 2** | <LOD |  | <LOD | <LOD | <LOD | <LOD | <LOD | <LOD | <LOD | <LOD | <LOD | 0.387 | | 0.221 | <LOD | <LOD | <LOD | 0.532 | 0.193 | 0.256 | <LOD | <LOD | <LOD | <LOD |
| **Blank 3** | <LOD |  | <LOD | <LOD | <LOD | <LOD | <LOD | <LOD | <LOD | <LOD | <LOD | 0.386 | | 0.379 | <LOD | <LOD | <LOD | 0.434 | 0,192 | 0,247 | <LOD | <LOD | <LOD | <LOD |
| **Blank 4** | <LOD |  | <LOD | <LOD | <LOD | <LOD | <LOD | <LOD | <LOD | <LOD | <LOD | 0.378 | | 0.379 | <LOD | <LOD | <LOD | 0.434 | 0.191 | 0.247 | <LOD | <LOD | <LOD | <LOD |
| **Blank 5** | <LOD |  | <LOD | <LOD | <LOD | <LOD | <LOD | <LOD | <LOD | <LOD | <LOD | 0.378 | | 0.230 | <LOD | <LOD | <LOD | 0.521 | 0.192 | 0.258 | <LOD | <LOD | <LOD | <LOD |
| **Blank 6** | <LOD |  | <LOD | <LOD | <LOD | <LOD | <LOD | <LOD | <LOD | <LOD | <LOD | 0.370 | | 0.380 | <LOD | <LOD | <LOD | 0.521 | 0.192 | 0.251 | <LOD | <LOD | <LOD | <LOD |
| **Blank 7** | <LOD |  | <LOD | <LOD | <LOD | <LOD | <LOD | <LOD | <LOD | <LOD | <LOD | 0.371 | | 0.270 | <LOD | <LOD | <LOD | 0.449 | 0.190 | 0.238 | <LOD | <LOD | <LOD | <LOD |
| **Blank 8** | <LOD |  | <LOD | <LOD | <LOD | <LOD | <LOD | <LOD | <LOD | <LOD | <LOD | 0.389 | | 0.380 | <LOD | <LOD | <LOD | 0.497 | 0.191 | 0.233 | <LOD | <LOD | <LOD | <LOD |
| **Blank 9** | <LOD |  | <LOD | <LOD | <LOD | <LOD | <LOD | <LOD | <LOD | <LOD | <LOD | 0.382 | | 0.380 | <LOD | <LOD | <LOD | 0.477 | 0.191 | 0.23 | <LOD | <LOD | <LOD | <LOD |
| **Blank 10** | <LOD |  | <LOD | <LOD | <LOD | <LOD | <LOD | <LOD | <LOD | <LOD | <LOD | 0.381 | | 0.381 | <LOD | <LOD | <LOD | 0.531 | 0.189 | 0.244 | <LOD | <LOD | <LOD | <LOD |
| **Blank 11** | <LOD |  | <LOD | <LOD | <LOD | <LOD | <LOD | <LOD | <LOD | <LOD | <LOD | 0.382 | | 0.251 | <LOD | <LOD | <LOD | 0.530 | 0.187 | 0.243 | <LOD | <LOD | <LOD | <LOD |
| **Blank 12** | <LOD |  | <LOD | <LOD | <LOD | <LOD | <LOD | <LOD | <LOD | <LOD | <LOD | 0.3817 | | 0.381 | <LOD | <LOD | <LOD | 0.518 | 0.192 | 0.249 | <LOD | <LOD | <LOD | <LOD |
| **Blank 13** | <LOD |  | <LOD | <LOD | <LOD | <LOD | <LOD | <LOD | <LOD | <LOD | <LOD | 0.378 | | 0.359 | <LOD | <LOD | <LOD | 0.400 | 0.197 | 0.29 | <LOD | <LOD | <LOD | <LOD |
| **Blank 14** | <LOD |  | <LOD | <LOD | <LOD | <LOD | <LOD | <LOD | <LOD | <LOD | <LOD | 0.368 | | 0.380 | <LOD | <LOD | <LOD | 0.472 | 0.189 | 0.262 | <LOD | <LOD | <LOD | <LOD |
| **%RSD** |  |  |  |  |  |  |  |  |  |  |  | 1.99 | | 18.7 |  |  |  | 9.17 | 1.01 | 5.90 |  |  |  |  |
| **Average** |  |  |  |  |  |  |  |  |  |  |  | 0.380 | | 0.337 |  |  |  | 0.489 | 0.192 | 0.251 |  |  |  |  |

**Table S8** Workflow Preparation Blanks Concentration in (pg/µL)

|  | **PFBA** | **PFPeA** | **PFHxA** | **PFHpA** | **PFOA** | **PFNA** | **PFDA** | **PFUnA** | **PFDoA** | **PFTrDA** | **PFTeDA** | **PFBS** | **PFPeS** | **PFHxS** | **PFHpS** | **PFOS** | **PFNS** | **PFDS** | **PFDoS** | **4:2 FTS** | **6:2 FTS** | **8:2 FTS** |
| --- | --- | --- | --- | --- | --- | --- | --- | --- | --- | --- | --- | --- | --- | --- | --- | --- | --- | --- | --- | --- | --- | --- |
| **Preparation Blank 1** | 8.951 | 1.605 | <LOD | <LOD | <LOD | <LOD | <LOD | <LOD | 0.548 | <LOD | 0.399 | 0.277 | <LOD | <LOD | <LOD | 0.573 | 0.190 | 0.229 | <LOD | 0.795 | <LOD | <LOD |
| **Preparation Blank 2** | 8.645 | 1.624 | <LOD | <LOD | <LOD | <LOD | <LOD | <LOD | 0.550 | <LOD | 0.394 | 0.382 | <LOD | <LOD | <LOD | 0.490 | 0.188 | 0.243 | <LOD | 0.742 | <LOD | <LOD |
| **Preparation**  **Blank 3** | 8.218 | 1.713 | <LOD | <LOD | <LOD | <LOD | <LOD | <LOD | 0.552 | <LOD | 0.389 | 0.381 | <LOD | <LOD | <LOD | 0.415 | 0.175 | 0.234 | <LOD | 0.622 | <LOD | <LOD |
| **Preparation Blank 4** | 8.237 | 1.691 | <LOD | <LOD | <LOD | <LOD | <LOD | <LOD | 0.547 | <LOD | 0.377 | 0.250 | <LOD | <LOD | <LOD | 0.413 | 0.186 | 0.242 | <LOD | 0.622 | <LOD | <LOD |
| **Preparation**  **Blank 5** | 7.961 | 1.644 | <LOD | <LOD | <LOD | <LOD | <LOD | <LOD | 0.549 | <LOD | 0.337 | 0.381 | <LOD | <LOD | <LOD | 0.388 | 0.218 | 0.214 | <LOD | 0.836 | <LOD | <LOD |
| **Preparation**  **Blank 6** | 7.866 | 1.648 | <LOD | <LOD | <LOD | <LOD | <LOD | <LOD | 0.548 | <LOD | 0.387 | 0.365 | <LOD | <LOD | <LOD | 0.410 | 0.194 | 0.225 | <LOD | 0.854 | <LOD | <LOD |
| **Preparation Blank 7** | 7.973 | 1.641 | <LOD | <LOD | <LOD | <LOD | <LOD | <LOD | 0.547 | <LOD | 0.384 | 0.282 | <LOD | <LOD | <LOD | 0.490 | 0.154 | 0.253 | <LOD | 0.895 | <LOD | <LOD |
| **Preparation Blank 8** | 6.820 | 1.319 | <LOD | <LOD | <LOD | <LOD | <LOD | <LOD | 0.561 | <LOD | 0.370 | 0.389 | <LOD | <LOD | <LOD | 0.583 | 0.174 | 0.253 | <LOD | 0.756 | <LOD | <LOD |
| **Preparation Blank 9** | 7.833 | 1.622 | <LOD | <LOD | <LOD | <LOD | <LOD | <LOD | 0.447 | <LOD | 0.386 | 0.339 | <LOD | <LOD | <LOD | 0.586 | 0.217 | 0.241 | <LOD | 0.743 | <LOD | <LOD |
| **Preparation Blank 10** | 7.811 | 1.554 | <LOD | <LOD | <LOD | <LOD | <LOD | <LOD | 0.548 | <LOD | 0.367 | 0.382 | <LOD | <LOD | <LOD | 0.218 | 0.142 | 0.242 | <LOD | 0.678 | <LOD | <LOD |
| **Average** | 8.032 | 1.606 |  |  |  |  |  |  | 0.540 |  | 0.379 | 0.343 |  |  |  | 0.457 | 0.184 | 0.238 |  | 0.754 |  |  |
| **%RSD** | 7.06 | 7.26 |  |  |  |  |  |  | 6.08 |  | 4.70 | 15.46 |  |  |  | 24.89 | 13.14 | 5.17 |  | 12.44 |  |  |

**Equations for the determination of uncertainty**

**Equation S1:**

$s^{2}\boldsymbol{=}\frac{\sum{(x_{i}-\bar{x} )}^{2}}{n-1}$ (S1)

where $x_{i}$ concentration determined for *i*^th^ replicate, $\bar{x}$ is the sample mean and *n* is the number of replicates.

**Equation S2:**

$u_{matrix = \frac{{\%RSD}_{x}}{n}}$ (S2)

where *%RSD* is the percent relative standard deviation of the determined sample concentration and *n* is the number of replicates

**Equation S3:**

$u_{recovery = \frac{{SD}_{rec}}{n_{rec}}}$ (S3)

where *SD_std_* is the standard deviation of the recoveries obtained for the target compound in the spiked for the number of replicates, *n_rec._*

**Equation S4:**

$u_{prep. blaks}\boldsymbol{=}\frac{\sum{(b_{i}-\bar{b} )}^{2}}{n_{b}-1}$ **(S4)**

where *bi* is the concentration determined for each target compound in the preparation blank replicates, *n_b_* and $\bar{b}$ is the mean.

**Table S9** Method Validation Table

| **Compound** | **R^2^** | | **LOD±%RSD (pg/g)** | **LOQ±%RSD (pg/g)** | **%RELATIVE Recovery ± %RSD** | | | | | |
| --- | --- | --- | --- | --- | --- | --- | --- | --- | --- | --- |
|  | **Calibration Range 1** | **Calibration Range 2** |  |  | **Paperboard (ng/g)** | | | **Paper  (ng/g)** | | |
|  |  |  |  |  | **10** | **20** | **100** | **10** | **20** | **100** |
| **PFBS** | 0.9875 | 0.9959 | 4.896±7.30 | 16.319±7.30 | 89±6.5 | 100±2.4 | 101±2.6 | 98±1.1 | 95±3.4 | 88±2.8 |
| **PFHxA** | 0.9959 | 0.9846 | 4.891±8.85 | 16.302±8.85 | 55±4.7 | 82±5.2 | 87±3.0 | 79±2.0 | 80±1.1 | 88±1.1 |
| **PFPeS** | 0.9861 | 0.9920 | 8.746±6.60 | 29.155±6.60 | 97±2.6 | 98±1.0 | 102±1.4 | 105±1.2 | 103±2.1 | 90±4.6 |
| **PFHxS** | 0.9882 | 0.9982 | 4.757±7.81 | 15.857±7.81 | 85±3.4 | 99±5.4 | 91± 1.9 | 103±1.9 | 102±1.6 | 101±2.3 |
| **PFHpA** | 0.9840 | 0.9919 | 2.898±3.22 | 9.661±3.22 | 69±1.9 | 70±1.6 | 109±3.9 | 99±1.0 | 97±2.0 | 89±1.2 |
| **PFOA** | 0.9887 | 0.9912 | 2.346±5.013 | 7.821±5.013 | 139±1.3 | 116±3.6 | 130±2.9 | 98±1.0 | 105±2.9 | 124±1.3 |
| **PFHpS** | 0.9875 | 0.9956 | 4.458±5.19 | 14.861±5.19 | 104±3.1 | 100±5.2 | 100±1.3 | 103±2.3 | 102±2.2 | 96±2.7 |
| **PFNA** | 0.9903 | 0.9930 | 4.048±5.48 | 13.495±5.48 | 92±1.5 | 93±4.7 | 103±1.7 | 98±9.8 | 96±2.9 | 97±1.1 |
| **PFOS** | 0.9855 | 0.9916 | 3.037±10.20 | 10.124±10.20 | 91±2.2 | 96±1.5 | 91±2.5 | 99±4.4 | 100±2.0 | 97±3.0 |
| **PFDA** | 0.9935 | 0.9930 | 1.465±3.90 | 4.885±3.90 | 92±1.7 | 90±2.7 | 97±1.9 | 111±1.8 | 119±4.5 | 111±2.3 |
| **PFNS** | 0.9930 | 0.9936 | 4.497±5.26 | 14.991±5.26 | 100±4.6 | 102±1.1 | 102±2.6 | 103±5.6 | 103±1.8 | 105±1.4 |
| **PFDS** | 0.9992 | 0.9922 | 3.667±1.54 | 12.222±1.54 | 80±1.5 | 73±1.4 | 75±1.1 | 76±2.5 | 75±2.1 | 79±1.8 |
| **PFUnA** | 0.9917 | 0.9914 | 10.067±7.55 | 33.557±7.55 | 95±3.2 | 97±1.0 | 104±1.0 | 77±3.8 | 76±7.5 | 79±2.6 |
| **PFDoA** | 0.9968 | 0.9968 | 5.097±5.98 | 16.991±5.98 | 102±1.3 | 102±2.9 | 103±3.6 | 89±5.2 | 92±3.2 | 87±3.4 |
| **PFDoS** | 0.9990 | 0.9928 | 3.925±5.39 | 13.082±5.39 | 78±1.7 | 86±1.1 | 83±1.9 | 78±1.7 | 84±2.4 | 83±2.7 |
| **PFTrDA** | 0.9959 | 0.9919 | 5.700±6.00 | 19.002±6.00 | 77±3.9 | 80±1.9 | 106±3.0 | 90±1.1 | 81±1.9 | 93±2.0 |
| **PFTeDA** | 0.9916 | 0.9959 | 4.329±9.85 | 14.429±9.85 | 84±1.5 | 88±2.3 | 88±2.3 | 86±3.6 | 84±7.5 | 88±1.3 |
|  |  |  |  |  | **Paperboard (ng/g)** | | | **Paper (ng/g)** | | |
|  |  | |  |  | **20** | **40** | **200** | **20** | **40** | **200** |
| **PFPeA** | 0.9922 | 0.9968 | 11.177±6.55 | 37.258±6.55 | 85±1.1 | 101±2.2 | 104±1.2 | 87±1.1 | 99±3.2 | 81±2.9 |
|  |  | |  |  | **40** | **80** | **400** | **40** | **80** | **400** |
| **PFBA** | 0.9922 | 0.9938 | 53.069±8.98 | 176.899±8.98 | 111±2.7 | 105±3.1 | 103±1.3 | 103±1.9 | 89±3.4 | 106±3.5 |
| **4:2 FTS** | 0.9889 | 0.9985 | 38.935±11.11 | 129.782±11.11 | 98±2.8 | 109±2.4 | 105±4.0 | 76±3.6 | 83±3.1 | 113±2.1 |
| **6:2 FTS** | 0.9898 | 0.9918 | 50.968±9.68 | 169.894±9.68 | 226±1.4 | 198±2.1 | 211±3.2 | 190±4.1 | 196±3.9 | 197±2.2 |
| **8:2 FTS** | 0.9938 | 0.9977 | 15.378±9.06 | 51.259±9.06 | 97±2.0 | 102±2.0 | 111±2.8 | 103±1.9 | 103±2.4 | 110±3.1 |

**Table S10** Categorical box and whisker plots category definitions

| **Stage** | | **Site** | | **Paper Recycling Grade** | |
| --- | --- | --- | --- | --- | --- |
| **Description** | **Assigned Number** | **Description** | **Assigned Number** | **Description** | **Assigned Number** |
| Pre-Consumer | 1 | Domestic waste | 1 | Cartonboard | 1 |
| Retail | 2 | Recycling facilities | 2 | Newsprint | 2 |
| Post-Consumer | 3 | Solid waste sites | 3 | Corrugated board | 3 |
|  |  | Waste Pickers | 4 | Black & white paper | 4 |
|  |  | Paper Mills | 5 | Magazine | 5 |
|  |  | Corrugators | 6 | Unconverted pre-consumer board | 6 |
|  |  | Retail | 7 | Corrugated pre-consumer | 7 |
|  |  |  |  | Coloured paper | 8 |

**Fig. S1** PFBA Box and Whisker Plot

**Fig. S2** PFPeA Box and Whisker Plot

**Fig. S3** PFBS Box and Whisker Plot

**Fig. S4** 4:2 FTS Box and Whisker Plot


**Fig. S5** PFHxA Box and Whisker Plot

**Fig. S6** PFHpA Box and Whisker Plot


**Fig. S7** PFPeS Box and Whisker Plot

**Fig. S8** PFHxS Box and Whisker Plot


**Fig. S9** 6:2 FTS Box and Whisker Plot

**Fig. S10** PFHpS Box and Whisker Plot


**Fig. S11** PFOA Box and Whisker Plot

**Fig. S12** PFNA Box and Whisker Plot

**Fig. S13** PFOS Box and Whisker Plot


**Fig. S14** 8:2 FTS Box and Whisker Plot


**Fig. S15** PFNS Box and Whisker Plot


**Fig. S16** PFDA Box and Whisker Plot


**Fig. S17** PFDS Box and Whisker Plot


**Fig. S18** PFUnA Box and Whisker Plot


**Fig. S19** PFDoA Box and Whisker Plot

**Fig. S20** PFDoS Box and Whisker Plot


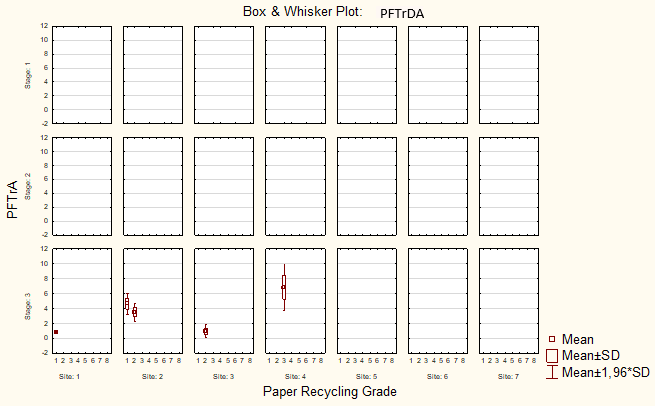


**Fig. S21** PFTrDA Box and Whisker Plot


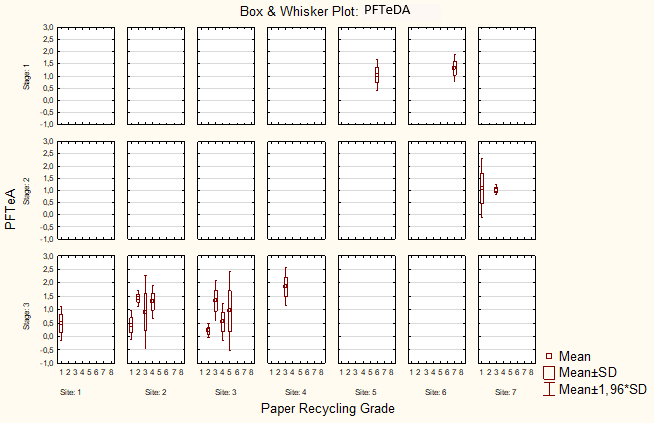

**Fig. S22** PFTeDA Box and Whisker Plot

**Table S11** Detected concentrations of PFCAs ± U (ng/g)

|  | **Description** | **PFBA** | **PFPeA** | **PFHxA** | **PFHpA** | **PFDA** | **PFDoA** | **PFUnA** | **PFTrDA** | **PFTeDA** | **PFNA** | **PFOA** |
| --- | --- | --- | --- | --- | --- | --- | --- | --- | --- | --- | --- | --- |
| Pre-Consumer | Paperboard | 0 | 0 | <LOD | <LOD | <LOD | 4.14±0.36 | <LOD | <LOD | 0 | <LOD | <LOD |
|  | Paperboard | 2.43±0.9 | 0 | 2.87±0.9 | <LOD | <LOD | 51.66±5.3 | <LOD | <LOD | 0.76±0.3 | 0.41±0.2 | <LOD |
|  | Paperboard | 46.81±5.6 | 0 | 1.48±0.9 | <LOD | 0.58±0.2 | 2.05±0.3 | <LOD | <LOD | 1.33±0.1 | 0.55±0.2 | <LOD |
|  | Corrugated box | 27.81±3.1 | 0 | 16.41±2.5 | <LOD | 0.54±0.2 | 26.02±3.2 | <LOD | <LOD | 1.03±0.3 | <LOD | <LOD |
|  | Corrugated fast food box | 16.02±1.9 | 0 | 2.01±0.1 | <LOD | 0.05±0.02 | 4.17±0.4 | <LOD | <LOD | 1.09±0.3 | <LOD | <LOD |
| Retail | Tea box | 25.42±1.6 | 0 | 2.36±0.3 | <LOD | 0.35±0.3 | 12.44±0.2 | 0.58±0.2 | <LOD | 1.54±0.1 | 27.20±2.8 | 0.25±0.1 |
|  | Chocolate box | 50.65±12.6 | 0 | 156.90±8.6 | 2.26±10.6 | 0.83±0.2 | 3.11±0.9 | <LOD | <LOD | 1.07±0.4 | 5.89±0.9 | 0.37±0.2 |
|  | Toy box | 25.19±1.1 | 0 | 9.43±1.2 | <LOD | 1.11±0.5 | 10.64±1.4 | <LOD | <LOD | 0 | <LOD | 2.4±0.6 |
|  | Electronics box | 60.99±2.4 | 0 | 6.85±1.3 | <LOD | 1.67±0.2 | 6.02±0.2 | 0.05±9.91 | <LOD | 0.94±0.1 | 9.07±0.4 | 2.60±0.4 |
|  | Noodles box | 13.91±1.8 | 0 | 1.46±0.2 | 1.62±0.6 | 0.76±0.3 | 7.88±0.3 | <LOD | <LOD | 1.14±0.4 | 1.77±0.2 | 0 |
|  | Electronics box | 14.51±2.0 | 0 | 4.14±1.9 | <LOD | <LOD | 14.17±0.9 | <LOD | <LOD | 0.28±0.4 | 0.09±0.05 | <LOD |
| Household | Fast-food container | 47.72±3.4 | 0 | 13.09±2.1 | 20.62±2.8 | 5.59±0.5 | 4.13±0.4 | <LOD | <LOD | 0.21±0.1 | <LOD | <LOD |
|  | Egg carton | 14.27±1.2 | 4.71±0.7 | 7.92±2.6 | 8.90±1.3 | <LOD | 3.65±0.3 | <LOD | 0.88±0.4 | 0.04±0.02 | 0.89±0.4 | <LOD |
|  | Paper bag | 17.22±1.1 | 0 | 7.36±0.9 | 4.51±0.6 | 0.54±0.2 | 8.12±0.5 | <LOD | <LOD | 1.08±0.4 | 0.30±0.1 | <LOD |
|  | Infant bath packaging | 15.64±4.7 | 0 | 0.90±0.3 | <LOD | <LOD | 9.90±2.0 | <LOD | <LOD | 0.04±0.02 | 2.49±0.3 | <LOD |
|  | Banana box | 15.75±1.1 | 0.84±0.1 | 3.23±0.9 | <LOD | <LOD | 24.72±0.6 | <LOD | <LOD | 0.21±0.08 | 3.96±0.8 | <LOD |
|  | Battery box | 21.46±3.8 ( | 0 | 6.38±1.1 | <LOD | <LOD | 2.84±0.8 | <LOD | <LOD | 0.06±0.03 | <LOD | <LOD |
| Recycling Site | Newspaper | 48.78±2.6 | 14.65±1.6 | 5.31±1.7 | 4.63±0.3 | <LOD | 13.72±1.0 | <LOD | 3.50±0.8 | 1.28±0.4 | 3.73±0.6 | <LOD |
|  | Newspaper insert | 2.77±0.6 | 0 | <LOD | <LOD | <LOD | 3.08±0.2 | <LOD | <LOD | 0 | 0.70±0.2 | <LOD |
|  | Avocado box | 10.08±1.2 | 0 | <LOD | <LOD | <LOD | 1.68±7.7 | <LOD | <LOD | 0.30±0.2 | <LOD | <LOD |
|  | Wine box | 24.59±1.5 | 0 | 2.91±0.9 | 4.31±0.9 | 4.61±0.4 | 18.5±0.2 | 0.41±0.2 | <LOD | 1.05±0.3 | 4.63±2.5 | 0.53±0.3 |
|  | Coffee box | 10.17±2.9 | 0 | 41.66±1.8 | <LOD | <LOD | 58.30±0.5 | <LOD | <LOD | 0 | <LOD | 1.54±0.5 |
|  | Tequila box | 33.12±1.2 | 41.54±2.0 | 4.66±1.0 | 35.40±4.3 | 2.85±0.6 | 15.42±0.9 | <LOD | 4.37±1.2 | 0.20±0.2 | 20.67±2.7 | <LOD |
|  | Cereal box | 0 | 0 | <LOD | <LOD | <LOD | 0 | <LOD | <LOD | 0.06±0.05 | <LOD | <LOD |
|  | Paper bag | 32.79±1.8 | 0 | 6.98±2.9 | 1.44±0.8 | 0.33±0.2 | 15.26±8.8 | 0.13±0.2 | <LOD | 0 | 15.23±1.3 | 1.31±0.5 |
|  | Colouring sheet | 10.62±0.6 | 0 | <LOD | <LOD | 0.45±0.2 | 0 | <LOD | <LOD | 1.29±0.4 | <LOD | 14.26±0.7 |
|  | Colouring box cover | 12.62±0.7 | 0.37±0.2 | <LOD | <LOD | <LOD | 0 | <LOD | <LOD | 0 | <LOD | <LOD |
| Solid Waste Site | Cookware box | 8.10±4.4 | 0 | 0.96±0.2 | <LOD | 0.09±0.1 | 4.99±0.1 | <LOD | <LOD | 0.93±0.1 | 27.72±2.8 | 0.60±0.3 |
|  | Brown box | 22.25±1.9 | 0 | 0.19±0.1 | 2.39±0.5 | 0.12±0.01 | 8.52±0.4 | <LOD | <LOD | 1.04±0.1 | 14.54±2.43 | 0.29±0.1 |
|  | Magazine | 27.97±2.3 | 0 | 1.26±0.4 | 1.09±0.5 | 0.37±0.3 | 3.27±0.6 | 1.31±0.3 | <LOD | 1.57±0.2 | <LOD | <LOD |
|  | Magazine | 30.96±0.3 | 0 | <LOD | 9.73±1.0 | <LOD | 8.57±0.6 | <LOD | <LOD | 0.10±0.1 | 0.56±0.2 | 6.96±1.4 |
|  | School Newsletter | 0 | 0 | <LOD | <LOD | <LOD | 0.05±0.05 | <LOD | <LOD | <LOQ | <LOD | <LOD |
|  | Newspaper | 11.03±3.6 | 0.63±0.5 | <LOD | <LOD | <LOD | 12.97±0.8 | <LOD | <LOD | 0 | 3.56±0.6 | <LOD |
|  | Newspaper | 28.61±7.4 | 25.84±1.0 | 4.46±0.9 | 10.20±2.3 | <LOD | 6.03±0.5 | <LOD | 0.8±0.5 | 0.21±0.3 | 8.11±1.1 | 3.15±0.5 |
|  | Corrugated food box | 33.94±1.0 | 0 | 1.53±0.7 | 10.14±0.7 | <LOD | 13.37±1.6 | <LOD | <LOD | 1.68±0.3 | <LOD | <LOD |
| Waste Pickers | Snacks box | 16.31±1.8 | 0 | 28.04±4.3 | <LOD | 0.47±0.3 | 6.51±3.8 | <LOD | <LOD | 1.53±0.1) | <LOD | <LOD |
|  | Coffee box | 6.62±1.0 | 0 | 7.67±0.3 | <LOD | 0.12±0.2 | 14.18±0.9 | <LOD | <LOD | 1.90±0.3 | 0.54±0.2 | 0.33±0.3 |
|  | Board | 60.56±1.3 | 1.13±0.2 | 3.35±0.2 | <LOD | <LOD | 0 | <LOD | <LOD | 0 | <LOD | <LOD |
|  | Corrugated food box | 61.51±2.8 | 70.37±3.1 | 19.58±2.1 | 47.29±5.2 | 38.67±0.2 | 4.30±0.9 | 6.61±0.2 | 6.8±1.7 | 1.93±0.4 | 1.50±0.4 | <LOD |

**Table S12** Detected concentrations of PFSAs ± U (ng/g)

|  | **Description** | **PFBS** | **PFOS** | **PFPeS** | **PFHxS** | **PFHpS** | **4:2FTS** | **6:2FTS** | **8:2FTS** | **PFNS** | **PFDS** | **PFDoS** |
| --- | --- | --- | --- | --- | --- | --- | --- | --- | --- | --- | --- | --- |
| Pre-Consumer | Paperboard | 0 | 0 | 1.57±0.2 | 9.96±1.0 | <LOD | 0 | 23.03±9.2 | <LOD | 0 | 0 | <LOD |
|  | Paperboard | 0 | 3.22±0.8 | <LOD | 5.16±0.8 | <LOD | 4.27±1.8 | 26.33±13.0 | <LOD | 0 | 0 | <LOD |
|  | Paper | 0 | 0 | <LOD | <LOD | <LOD | 0 | 22.16±11.2 | 0.46±0.2 | 0 | 0 | <LOD |
|  | Corrugated box | 0 | 0 | 1.46±0.2 | 19.87±2.1 | <LOD | 6.17±2.7 | 2.00±1.4 | <LOD | 0 | 0 | <LOD |
|  | Corrugated fast food box | 0.18±0.1 | 0 | <LOD | 11.06±0.1 | <LOD | 0 | 53.83±16.0 | <LOD | 0 | 0 | <LOD |
| Retail | Tea box | 1.50±0.6 | 0 | 2.11±0.1 | 1.39±0.7 | <LOD | 1.17±0.99 | 57.21±18.8 | 2.89±0.8 | 0 | 0 | <LOD |
|  | Chocolate box | 0 | 0 | 1.70±0.4 | <LOD | <LOD | 0 | 11.51±4.5 | <LOD | 0.78±0.1 | 0 | <LOD |
|  | Toy box | 0 | 0 | 1.22±0.5 | <LOD | 2.47±0.2 | 1.40±0.5 | 50.71±23.4 | <LOD | 0 | 16.68±1.2 | <LOD |
|  | Electronics box | 2.04±0.3 | 0 | 0.42±0.3 | 1.26±0.4 | 1.94±0.5 | 7.30±3.3 | 49.84±15.1 | 0.41±0.2 | 0 | 4.35±1.3 | 4.67±0.7 |
|  | Noodles box | 0 | 0.36±0.2 | 1.63±0.1 | <LOD | 1.18±0.1 | 5.04±0.3 | 5.46±5.9 | 6.14±1.7 | 0 | 17.58±3.2 | 3.88±0.5 |
|  | Electronics box | 0 | 0 | <LOD | 2.96±0.6 | <LOD | 10.50±1.8 | 7.30±5.10 | <LOD | 0 | 17.54±1.6 | <LOD |
| Household | Fast-food container | 0 | 44.36±1.6 | 0.51±0.1 | <LOD | 0.74±0.3 | 0 | <LOD | <LOD | 0 | 0 | <LOD |
|  | Egg carton | 3.43±0.2 | 5.46±0.6 | 9.10±0.7 | 13.06±0.7 | 0.40±0.1 | 42.35±5.9 | 10.10±1.5 | 0.62±0.2 | 0 | 1.16±0.1 | 0.70±0.4 |
|  | Paper bag | 0 | 0 | <LOD | 6.50±0.4 | <LOD | 0 | 80.88±22.5 | 8.66±0.3 | 0 | 0 | <LOD |
|  | Infant bath packaging | 0 | 2.09±0.7 | <LOD | 5.31±0.6 | <LOD | 7.90±1.4 | 32.23±13.3 | <LOD | 0 | 0 | <LOD |
|  | Banana box | 1.34±0.3 | 0.49±0.4 | 0.96±0.4 | 3.36±0.3 | 0.54±0.1 | 5.63±0.4 | 35.39%±12.9 | 4.87±0.3 | 0.06±0.1 | 0.89±0.1 | 0.76±0.5 |
|  | Battery box | 0 | 0 | <LOD | 2.12±0.3 | <LOD | 0 | 4.97±2.6 | <LOD | 0 | 0 | <LOD |
| Recycling Site | Newspaper | 1.70±0.1 | 0.99±0.1 | 3.24±0.4 | 16.85±2.2 | 0.25±0.1 | 31.12±6.6 | 249.33±42.7 | <LOD | 0 | 1.16±0.7 | <LOD |
|  | Newspaper insert | 1.27±0.1 | 4.95±0.6 | 2.08±0.1 | 1.27±0.6 | 1.11±0.1 | 17.09±2.9 | 36.44±9.7 | 2.70±0.4 | 0 | 0 | <LOD |
|  | Avocado box | 0 | 0 | <LOD | 19.81±0.2 | <LOD | 0 | <LOD | <LOD | 0 | 0 | <LOD |
|  | Wine box | 1.27±0.2 | 0 | <LOD | 25.34±2.2 | 0.02±0.1 | 2.04±0.7 | 14.44±7.6 | 9.34±0.5 | 0.30±0.1 | 0 | 1.98±0.5 |
|  | Coffee box | 0 | 0 | <LOD | 8.08±0.8 | <LOD | 0 | 28.04±11.7 | <LOD | 0 | 14.51±1.5 | <LOD |
|  | Tequila box | 6.22±0.3 | 0.72±0.3 | 28.40±11.5 | 21.94±1.8 | 7.19±0.2 | 54.85±0.3 | 354.51±88.1 | 11.33±1.0 | 0 | 3.06±18.7 | 1.14±0.6 |
|  | Cereal box | 0.40±0.2 | 1.13±0.2 | 1.28±0.2 | 1.22±0.3 | 0.16±0.1 | 4.60±0.9 | 2.45±2.1 | 2.43±0.9 | 0 | 0 | 0.72±0.5 |
|  | Paper bag | 0.46±0.2 | 0 | <LOD | 28.17±0.3 | 4.43±1.7 | 0 | <LOD | 2.80±0.5 | 0.38±0.1 | 1.11±0.1 | 0.60±0.4 |
|  | Colouring sheet | 0 | 0 | <LOD | <LOD | <LOD | 3.60±2.8 | <LOD | <LOD | 0 | 0 | <LOD |
|  | Colouring box cover | 0.38±0.2 | 0.80±0.2 | 1.05±0.7 | <LOD | 0.28±0.1 | 0.90±0.8 | <LOD | 0.17±0.2 | 0 | 0 | 0.66±0.5 |
| Solid Waste Site | Cookware box | 0 | 0 | <LOD | <LOD | <LOD | 0 | 68.83±26.6 | 1.50±0.3 | 0.18±0.1 | 1.24±0.4 | 1.44±0.4 |
|  | Brown box | 1.70±0.2 | 0 | <LOD | 0.68±0.5 | 1.13±0.1 | 0 | 24.19±12.6 | 1.32±0.2 | 0.17±0.1 | 0.47±0.1 | 0.68±0.5 |
|  | Magazine | 0 | 0 | <LOD | <LOD | <LOD | 0 | <LOD | <LOD | 0 | 0 | <LOD |
|  | Magazine | 0 | 0 | 12.21±0.4 | 23.82±1.4 | 5.09±0.6 | 33.65±3.3 | 22.78±10.5 | <LOD | 0 | 0 | <LOD |
|  | School Newsletter | 0 | 0 | <LOD | <LOD | <LOD | 2.52±2.8 | <LOD | <LOD | 0 | 0 | <LOD |
|  | Newspaper | 1.19±0.6 | 2.80±0.8 | 1.73±0.2 | 0.55±0.3 | 1.65±0.7 | 11.39±2.9 | 43.20±17.9 | 0.98±0.2 | 0 | 0.32±0.2 |  |
|  | Newspaper | 1.01±0.2 | 8.71±0.3 | 12.91±0.7 | 14.67±2.1 | 2.30±0.5 | 28.45±11.1 | 60.28±16.1 | 3.80±0.3 | 0 | 0 | 0.92±0.5 |
|  | Corrugated food box | 0.67±0.2 | 2.81±0.2 | <LOD | <LOD | <LOD | 14.87±4.2 | 9.38±6.0 | <LOD | 0.28±0.1 | 0 | <LOD |
| Waste Pickers | Snacks box | 0 | 0.17±0.2 | <LOD | 2.02±1.7 | 0.44±0.1 | 25.97±3.0 | 24.08±8.1 | <LOD | 0 | 0 | <LOD |
|  | Coffee box | 0 | 0 | 2.02±0.1 | <LOD | 0.19±0.1 | 0 | 3.45±2.4 | 2.49±0.2 | 0 | 0.32±0.3 | <LOD |
|  | Board | 1.82±0.2 | 0.43±0.3 | 1.58±0.2 | <LOD | 0.25±0.1 | 10.13±1.0 | 29.07±11.0 | 3.15±0.5 | 0 | 0.47±0.3 | 0.69±0.4 |
|  | Corrugated food box | 15.55±1.2 | 9.75±1.0 | 10.60±2.5 | 17.34±1.2 | 1.56±0.1 | 164.79±7.1 | 20.84±11.5 | 22.05±6.6 | 0.60±0.1 | 18.30±2.1 | 8.03±7.7 |


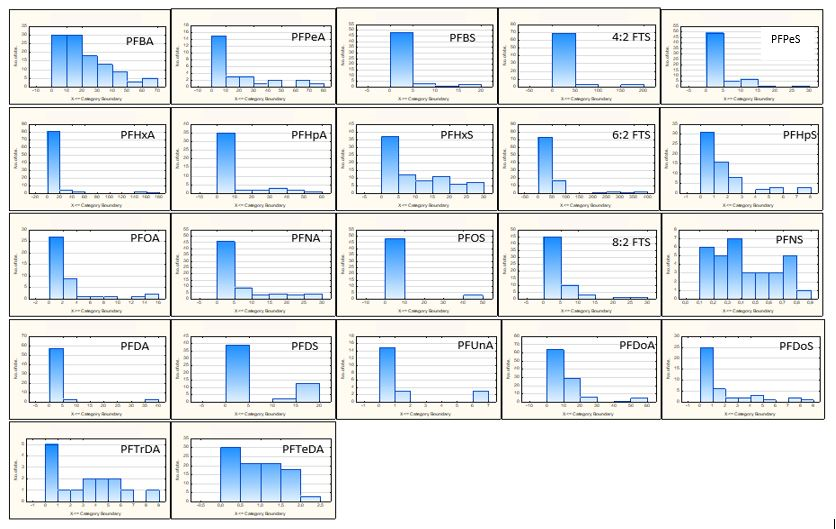
 **Fig. S23** Uncategorised Distribution plots

**References**

MacLennan MS, Ng D, Hope D (2019) Extraction of poly-and perfluorinated alkyl substances (PFAS) from solid matrices. Society of Ecotoxicology and Chemistry (SETAC) North America, 40th Annual Meeting, Toronto, Poster. https://doi.org/10.6084/m9.figshare.13557185.v

MacLennan MS, Ng D, Hope D (2021) Extraction of poly-and perfluorinated alkyl substances (PFAS) from soli. Pacific Rim Laboratories. Thermo Scientific Customer Application Note 73937

Silva P, Vorst K, Curtzwiler GW (2021) The effect of particle size reduction techniques on extraction and recovery of 16 PFAS in food-contact paper packaging matrices.
Thermo Scientific Application Note 65960. https://assets.thermofisher.com/TFS-Assets/CMD/Application-Notes/an-65960-lc-ms-pfas-food-packaging-matrices-an65960-en.pdf

Trier X, Granby K,and Christensen J H (2011) Polyfluorinated surfactants (PFS) in paper and board coatings for food packaging Environ Sci Pollut Res Int 18(7):1108-20. http://doi.org/10.1007/s11356-010-0439-3

Rasul SB, Monsur Kajal A, Khan AH (2017) Quantifying uncertainty in analytical measurements. J Bangladesh Acad Sci 41(2): 145–163
